# Long-term soil metal exposure impaired temporal variation in microbial metatranscriptomes and enriched active phages

**DOI:** 10.1186/s40168-018-0606-1

**Published:** 2018-12-13

**Authors:** Samuel Jacquiod, Inês Nunes, Asker Brejnrod, Martin A. Hansen, Peter E. Holm, Anders Johansen, Kristian K. Brandt, Anders Priemé, Søren J. Sørensen

**Affiliations:** 1 Section of Microbiology, University of Copenhagen, Universitetsparken 15, 2100 Copenhagen, Denmark; 2 Agroécologie, AgroSup Dijon, INRA, Univ Bourgogne Franche-Comté, 17 rue Sully, 21000 Dijon, France; 3 Present address: Microbe Technology Department, Novozymes A/S, Krogshoejvej 36, 2880 Bagsværd, Denmark; 4 Present address: Center for Basic Metabolic Research, University of Copenhagen, Blegdamsvej 3A, 2200 Copenhagen, Denmark; 5 Present address: Department of Plant and Environmental Sciences, University of Copenhagen, Thorvaldsensvej 40, 1871 Frederiksberg C, Denmark; 6 Department of Environmental Science, Aarhus University, Frederiksborgvej 399, 4000 Roskilde, Denmark

**Keywords:** Cu pollution, Metatranscriptomics, Temporality, Phages, Microbial adaptation, Soil functioning

## Abstract

**Background:**

It remains unclear whether adaptation and changes in diversity associated to a long-term perturbation are sufficient to ensure functional resilience of soil microbial communities. We used RNA-based approaches (16S rRNA gene transcript amplicon coupled to shotgun mRNA sequencing) to study the legacy effects of a century-long soil copper (Cu) pollution on microbial activity and composition, as well as its effect on the capacity of the microbial community to react to temporal fluctuations.

**Results:**

Despite evidence of microbial adaptation (e.g., iron homeostasis and avoidance/resistance strategies), increased heterogeneity and richness loss in transcribed gene pools were observed with increasing soil Cu, together with an unexpected predominance of phage mRNA signatures. Apparently, phage activation was either triggered directly by Cu, or indirectly via enhanced expression of DNA repair/SOS response systems in Cu-exposed bacteria. Even though total soil carbon and nitrogen had accumulated with increasing Cu, a reduction in temporally induced mRNA functions was observed. Microbial temporal response groups (TRGs, groups of microbes with a specific temporal response) were heavily affected by Cu, both in abundance and phylogenetic composition.

**Conclusion:**

Altogether, results point toward a Cu-mediated “decoupling” between environmental fluctuations and microbial activity, where Cu-exposed microbes stopped fulfilling their expected contributions to soil functioning relative to the control. Nevertheless, some functions remained active in February despite Cu, concomitant with an increase in phage mRNA signatures, highlighting that somehow, microbial activity is still happening under these adverse conditions.

**Electronic supplementary material:**

The online version of this article (10.1186/s40168-018-0606-1) contains supplementary material, which is available to authorized users.

## Background

Following industrial and agricultural revolutions, the fast development of our societies increased anthropogenic pressure on soils, notably via intensive farming and use of phytosanitary products, such as metal/metalloid derived pesticides. Due to their non-degradable nature, these compounds represent an extremely persistent pollution, often accumulating in specific environmental hotspots (e.g., soils and sediments), leaving microorganisms constantly exposed [1, 2]. As such, metal pollution qualifies as “press-type” disturbance, as opposed to “pulse-type” [3]. Depending on physicochemical conditions, metals may become bioavailable and toxic for microbes, which in return may establish resistance/tolerance mechanisms [4]. Cu-derived pesticides (e.g., Cu-sulfate) are commonly used in agriculture and wood-impregnation plants since the mid-eighteenth century [1], representing a well-studied metal contaminant in soils [5].

Long-term metal pollution may have contrasting effects on diversity of environmental microbial communities [1, 2, 6, 7]. While much is known about microbial tolerance toward metals, it remains unclear if mere adaptation is sufficient to guarantee functional recovery [8, 9] and ultimately maintain microbial contributions to soil ecosystem functioning [10]. This relation between biodiversity and ecosystem functioning relates to the “insurance hypothesis,” assuming that greater species richness prevents function decline through temporal variance buffering and increasing ecological performance [11]. Microbiomes may show differences in phylogenetic profiles, diversity levels, and gene abundance/expression, making it difficult to establish a general mechanistic understanding of how this may influence ecosystem functionality [12, 13]. In addition, temporal considerations when studying the ecology of environmental microbial communities are still lacking despite being crucial for establishing the missing links between diversity and function in microbial ecology [14]. This is particularly true for soils, as their functioning is tightly associated to environmental conditions such as temperature, water, and nutrient availability, with direct consequences on microbial communities [15–19]. Although the recent advances in mRNA sequencing now allow direct access to microbial expression profiles, soil metatranscriptomic studies focusing on environmental fluctuations linked to temporal aspects are still scarce [15]. Consequently, it remains unresolved if soil microbes coping with persistent pollution possess/express sufficient genetic diversity to maintain their functions under ever-fluctuating environmental conditions.

The experimental field located in Hygum (Denmark), displaying a century-long Cu concentration gradient (15–4000 mg Cu kg^−1^), allows to shed light on some of these aspects. Previous 16S rRNA gene transcript amplicon analysis on this site revealed an important “Cu-legacy” effect on soil prokaryotes overruling temporal fluctuations and defined as the sum of entangled direct/indirect effects of Cu on this ecosystem [1]. However, temporal and functional aspects related to the microbial communities copping with this long-term “press-type” stress were never studied. The present study aims at (i) bringing new knowledge on microbial adaptation mechanisms to persistent and contrasted Cu pollution concentrations, while (ii) deciphering how Cu has influenced the capacity of the soil microbial community to react to environmental fluctuations. We hypothesized that despite signs of adaptation due long-term exposure, microbes were still hampered by Cu, resulting in deep phylogenetic restructuring of active microorganisms reacting to environmental fluctuations, with deleterious consequences on soil functioning. We sequenced soil metatranscriptomes in this century-long Cu polluted site at three different contamination levels (control, semi-contaminated, hotspot) and for three contrasted time points over the course of a year (August 2013, February 2014, August 2014). For a more comprehensive picture, our mRNA results were combined with microbial respiration (MicroResp™), biomass (PLFA), and 16S rRNA gene transcript amplicon sequencing data from Nunes et al. [1] (data from February and August 2014). This time, analysis was done through the prism of environmental fluctuation, with new data from an additional sampling time point done in August 2013 (this study). To account for the strong Cu-legacy, we defined microbial temporal response groups (TRGs) to identify OTUs significantly responding to environmental fluctuations separately at each site. This approach was previously validated on perturbed environmental microbiomes to decipher effects of soil drought [17], water quality [20], as well as metal pollution in soil [1] and sediment [2]. This study brings a significant contribution to the current understanding of microbial community adaptation and temporal responses under long-term persistent pollution, highlighting an unexpected and puzzling increase of phage mRNA signatures.

## Results and discussion

### Cu-legacy on mRNA profiles

**Fig. 1:**
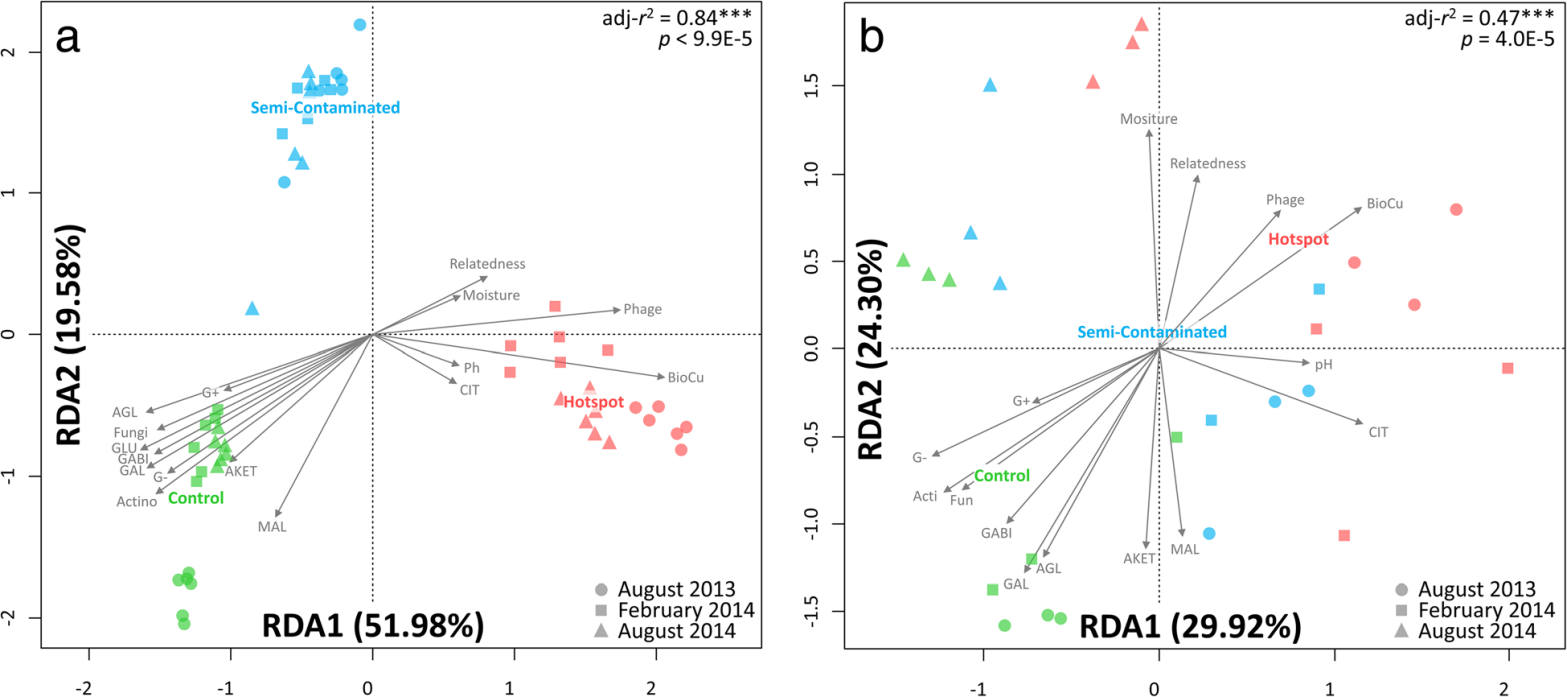
Redundancy analysis (RDA) on 16S rRNA transcript amplicon profiles (cDNA, *n* = 54, panel **a**) and metatranscriptomes (mRNA, *n* = 26, panel **b**). Ordination plots were constrained using a model with explanatory variables (gray arrows) using Bray-Curtis dissimilarly index (R package *vegan*, *capscale* function, adjusted *r*^2^ from model tested with 10,000 permutations). Explanatory variables include substrate respiration (MicroResp, D(+) galactose (GAL), l-malic acid (MAL), gamma amino butyric acid (GABI), n-acetyl glucosamine (AGL), D(+) Glucose (GLU), Alpha Ketogluterate (AKET) and Citric Acid (CIT), PLFA fractions (G+: Gram positive, G-: Gram negative, Acti: Actinobacteria, Fun: Fungi), soil pH and moisture, bioavailable Cu (BioCu), proportion of phage mRNA signatures, and phylogenetic relatedness (− 1* MPD *Z*-score). Plots display the first and second constrained components with percentage of explained variance in datasets by the model. The colored plot names are indicating their centroid location as discriminant factors in the model

Plots exposed to different Cu concentrations for a century had distinct microbial communities (Fig. 1a, adjusted *r*^2^ = 0.84, *p* = 9.9E−5) and mRNA profiles (Fig. 1b, adjusted *r*^2^ = 0.47, *p* = 1.0E−4). Cu concentration was the dominating factor, correlating with reduced substrate respiration (except for citrate), microbial biomass, phylogenetic diversity (Additional file 1: Table S1), and OTU/mRNA diversity (Additional file 1: Figure S1). Significantly higher amounts of carbon and nitrogen have accumulated at the hotspot (Additional file 1: Table S1), likely due to lower degradation rates of organic matter observed in this site [21]. Previous studies linked decrease in soil functioning with altered microbial community composition [22, 23]. Accordingly, we observed the diminution of specific phylogenetic groups in Cu-plots (Additional file 1: Table S2), including Gammaproteobacteria and Actinobacteria, both harboring well-described members involved in active degradation of soil organic matter [24]. Concomitantly, Cu selected for microbial communities with higher phylogenetic relatedness (MPD index, Fig. 1, Additional file 1: Table S1) and restrained functional diversity (Additional file 1: Figure S1), being dominated by Acidobacteria Gp1/3/16, Betaproteobacteria, and Nitrospira (Additional file 1: Table S2). This suggests that remaining Cu-selected microbes may engage in competition/antagonism, which is often observed between phylogenetic close and functionally redundant individuals sharing the same niche [25].

Despite achieving very satisfactory mRNA sequencing yields (Additional file 1: Table S3), with relatively low level of leftover rRNA sequences (7–17%), our annotation efficiency remained low (1–10%). Nevertheless, our results are in the same range compared to other soil metatranscriptomic reports [15, 26]. Indeed, database representation is a recurrent bottleneck for environmental microbial sequencing projects, leading to poor coverage and potential for wrong annotations [27]. Although the amount of annotated sequences may be artificially inflated by using several databases and lowering the stringency of annotation cut-off, we opted for a very conservative strategy by restricting our analysis to the database offering the best coverage (SEED database, Additional file 1: Table S3), with a reinforced annotation cut-off to minimize false discoveries rate [27]. Despite technical limitations and the conservative annotation threshold applied, many mRNA functions were significantly altered by Cu. The following section will focus on the most relevant features.

**Fig. 2:**
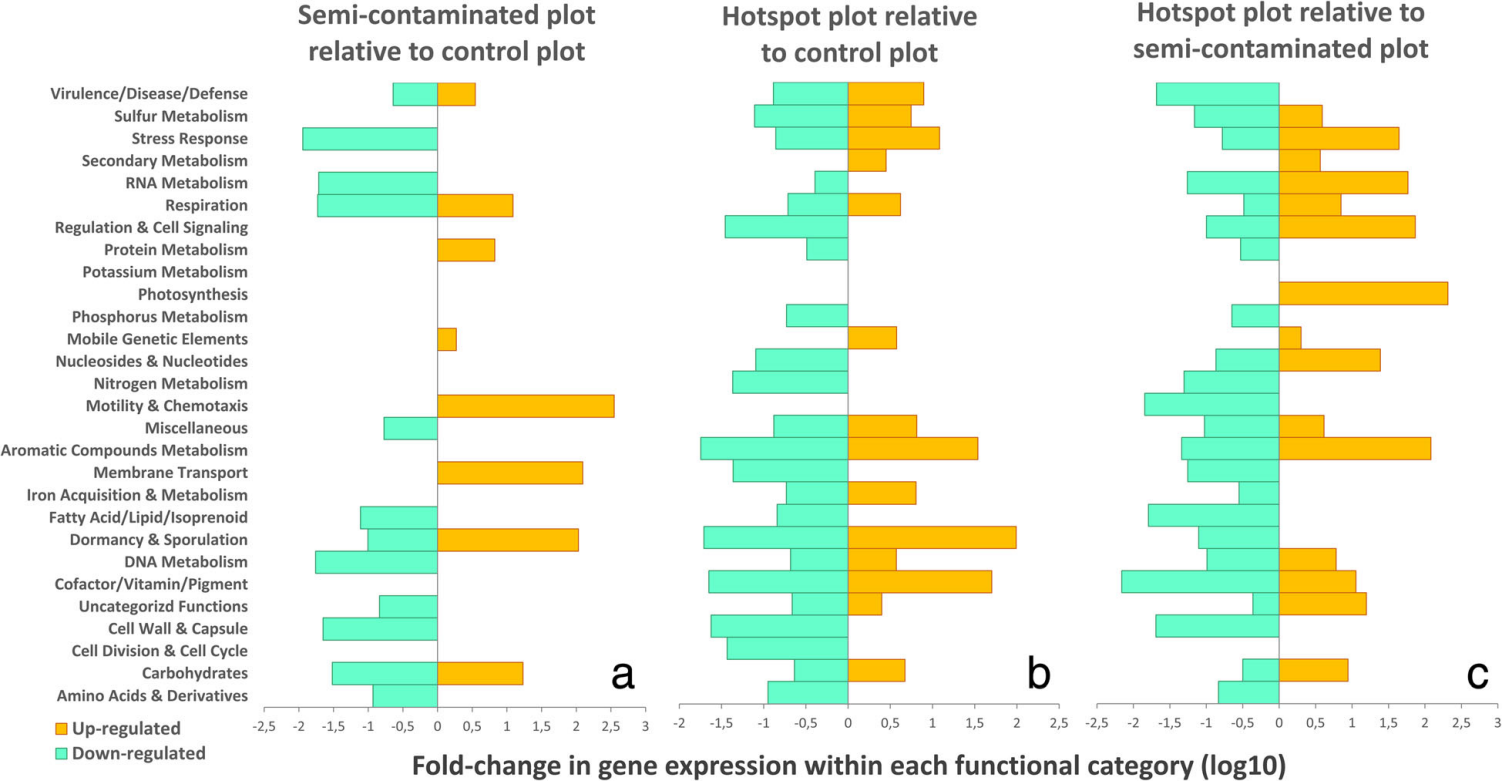
Metatranscriptomic pairwise ratio comparison of gene expression in the three plots. The figure shows the log10 up and downregulation ratios of functions within metabolic categories in Cu-plots relative to the control (panels **a** and **b**, downregulated = higher in the control; upregulated = higher in the copper plots), and between copper plots (panel **c**, downregulated = higher in the semi-contaminated; upregulated = higher in the hotspot). Absolute mRNA counts of functions significantly altered by Cu were extracted (nbGLM, LRT, FDR-corrected *p* < 0.05), summed per metabolic category, and respectively divided by either control plot values (panels **a** and **b**) or from the semi-contaminated plot (panel **c**) in order to define relative up and downregulation ratios. The displayed functional categories used are very large and encopass many subcategories and genes. Therefore, within a same category, some genes might be significantly upregulated while others might be downregulated

Analysis of gene expression ratio between sites revealed contrasted expression profiles, which were rarely associated with specific functional categories (Fig. 2). Indeed, different sets of genes within the same category were often either up or downregulated, especially when comparing the hotspot to the control and the semi-contaminated plots (Fig. 2b, c). This is a direct consequence of the diversity loss observed both at the phylogenetic and functional level, as many genes are either not expressed, or sometimes completely lost. As a consequence, focus will be given on upregulated functions in the copper plots. Both Cu-exposed communities upregulated similar functions related to “Virulence/Disease/Defense” mechanisms (log-10 fold changes relative to the control, Fig. 2a, b), including metal efflux pumps, multicopper oxydases, and metalloregulatory proteins to cope with Cu [28–30]. Despite this adaptation, microbes remained impacted by Cu, as revealed by activation of “Stress Response” (oxidative/detoxification mechanisms) and “DNA Metabolism” via repair systems in the hotspot, likely caused by Cu-induced DNA nicking [31, 32]. A metabolic shift occurred in the hotspot through upregulation of “Sulfur Metabolism” (organic/mineral assimilation) and “Iron Acquisition & Metabolism” (bacterioferritin, ferric siderophore transport, and iron-sulfur cluster protein synthesis). This is likely to compensate Cu-induced loss of essential iron-sulfur proteins involved in environmental sensing and gene expression regulation [33], leading to iron starvation and oxidative stress [34, 35]. The concomitant upregulation of “Cofactors/Vitamins” via heme-containing molecules (e.g., tetrapyrroles) and “Respiration” via electron donating/accepting reactions support this assertion, as mismetallation of metalloregulatory proteins by metal excess deregulate heme synthesis, leading to oxidative stress [35]. In nutrient-deprived anaerobic conditions, rescue of iron-sulfur clusters via Cu efflux systems was shown [36], highlighting the importance of maintaining iron homeostasis to support growth under adverse conditions. Specific “RNA Metabolism” features relating to tRNA methylation were upregulated, likely being another consequence of iron-sulfur clusters rescue. Indeed, tRNA methylation is important for sulfur/amino acid metabolism and protein biosynthesis [37], both supporting the replenishing of iron-sulfur clusters. Specific “Dormancy & Sporulation” functions were upregulated in both Cu-plots. Dormancy is a well-known bet-hedging strategy to survive unfavorable conditions [38, 39]. Conversely, metals are essential in sporulation regulation [40], but deleterious at high concentrations [41]. Most noticeable was the ~ 100-fold increase in *Bacillus* sporulation SpoVS system [42] in the hotspot. This suggests successful adaptation via endospore formation, as Bacillales (Firmicutes), was not affected by Cu (Additional file 1: Table S2), while dormant bacteria may accumulate 16S rRNA transcripts and be detectable via amplicon sequencing [43]. “Aromatic Compound Metabolism” upregulation (transport/degradation) likely indicates selection of microbial oligotrophs [20, 44] feeding on complex carbon molecules (e.g., humic-acids) that may have accumulated in the hotspot [21] within the increased total carbon content (Additional file 1: Table S1).

Despite adaptation similarities, different microbial functions were selected depending on Cu concentrations (Fig. 2c). In the semi-contaminated plot, specific upregulation of “Motility & Chemotaxis” (flagella), “Membrane Transport” (secretion systems III/VI/VIII) and “Cell-wall & Capsule” (capsular/extracellular polysaccharide/cell-wall biosynthesis) indicates selection of avoidance strategies relying either on negative chemotaxis [45], biofilm formation [46, 47], and/or physical barriers [4, 48], suggesting successful adaptation via adequate phenotypical tuning. Nevertheless, positive chemotaxis may also occur, indicating selection of microbes seeking contaminated niches [49]. The high local heterogeneity of Cu pollution may contribute to this observed increase in chemotaxis, as high Cu may form visible green precipitates in this site (Additional file 1: Figure S2). Contact-dependent stabbing structures are used to eliminate competitors (e.g., secretion systems VI [50]), supporting again the existence of antagonistic relationships that may prevail among Cu-selected competing bacteria. Upregulation of “Fatty Acids/Lipids & Isoprenoids” (biosynthesis/degradation) with “Cofactors/Vitamins/Pigments” (coenzyme A) functions likely indicates cell-wall/capsule-based resistance barriers via constituting components synthesis/recycling [4]. Additional upregulated cofactors biosynthesis (tetrapyroles/heme-structures) may indicate active metal chelating [51]. Overall, clear signs of diverse Cu avoidance/resistance/tolerance strategies and active metabolic processes were found in the semi-contaminated plot, indicating successful long-term adaptation. In the hotspot, functional regulation remained intriguing (Fig. 2c), with probable observations of indirect effects associated to the extreme Cu-legacy. Thus, “Photosynthesis” activation (photosystem I) may indicate selection of phototrophic microbes on the top soil due to poor plant coverage [1], and “Carbohydrate” upregulation (acetyl-coA, lactate/acetate/acetoin/butanediol fermentation) may indicate enhanced activity in anaerobic niches due to higher soil moisture content and compaction in this plot [1, 21]. Upregulation of “Regulation & Cell Signaling” (toxin-antitoxin systems) and “Potassium Metabolism” (efflux systems) certainly plays a protective role against adverse conditions directly caused by the Cu-legacy [52, 53]. “Secondary Metabolism” upregulation (peroxidase response) may play a protective role against metal-induced oxidative stress [54]. Specific “Respiration” functions activation (metallo-enzymes/electron carriers/formate hydrogenase) points toward active dihydrogen utilization as an energy source, supporting its relevance for microbial survival under adverse conditions [55]. Overall, results from the hotspot revealed exacerbated molecular/metabolic signs of deleterious exposure to extreme Cu dose.

### A Cu-mediated increase in phage mRNA

**Fig. 3:**
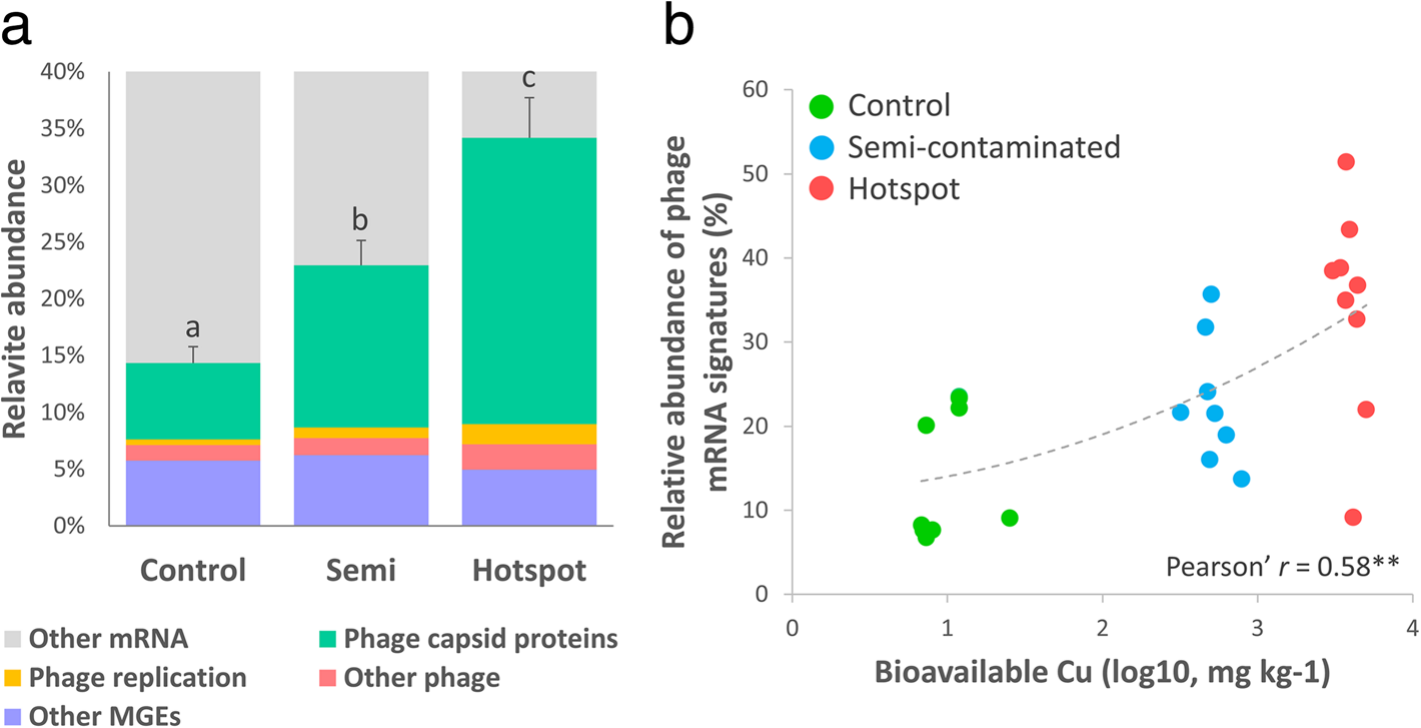
Panel **a** shows the relative abundance of functions within the “Mobile Genetic Elements” category (MGE) where phage mRNA signatures were detected among the metatranscriptomes of three plots (Control *n* = 9; Semi-contaminated *n* = 8; Hotspot *n* = 8). Statistical differences between time points in each plot were inferred with ANOVA (Tukey’s HSD post hoc test, *p* < 0.05). Letters are attributed in ascending order, “a” being the smallest average. Different letters indicate statistically significant differences (*p* < 0.05). Panel **b** shows the correlation between the relative abundance of phage mRNA signatures and bioavailable Cu in each samples from the three plots (Pearson’ *r* = 0.58, *p* = 1.2E−3)

An unexpected dominance of phage-related mRNA sequences was discovered in Cu-plots, accounting for ~ 30% of annotated mRNA in the hotspot (Fig. 3a). This represented a respective upregulation of 1.9- and 3.7-folds in the semi-contaminated and hotspot plots relative to the control (Fig. 2). The “Mobile Genetic Elements” category was dominated by active phage signatures (e.g., capsid synthesis and phage replication, Fig. 3a), correlating with bioavailable Cu (Pearson’ *r* = 0.58, *p* = 1.2E−3, Fig. 3b). Concomitantly, upregulation of central metabolic functions in Cu-plots (e.g., protein biosynthesis and nucleoside conversion, Fig. 2a, c) may reflect investments for novel phage-particle synthesis [56]. Unfortunately, our study design does not allow to resolve whether if these mRNA signatures originate from lytic or lysogenic phages, or perhaps both, neither their taxonomic affiliation nor their functional relevance. It is common place that both lytic and lysogenic strategies are present in complex environmental microbial communities [57], and lysogeny is thought to be favored under adverse conditions by enhancing both phage and host survival in soils [58]. Furthermore, phage cycles are rarely triggered spontaneously [59], as they often respond to external cues/stressors [57], including metal (e.g., Cu) [60]. One key molecular signal involved in phage-triggering is the host cell DNA repair mechanisms, so-called “SOS response” [61], which are activated upon DNA damage (e.g., caused by pollutants) [62]. As DNA-repair functions were significantly upregulated in Cu-plots (Fig. 2b, c), likely due to Cu-induced DNA nicking, we hypothesized that the SOS response could be a plausible triggering signal for this observed phage mRNA enrichment [63]. Soil moisture could also stand as a potential factor associated to phage activity. Indeed, as the hotspot had significantly higher moisture (Additional file 1: Table S1), their apparent success might be linked to associated benefits coming with water, such as better dispersal of phage particles [64]. Altogether, our observations suggest that bacteria-phage interactions may be either directly or indirectly linked to Cu pollution, which might hold a potential role for bacterial survival and functions under these adverse conditions, as already evidenced before [65]. Indeed, in our system, a metabolic shift occurred in communities subjected to elevated Cu, including specific upregulation of functions within “Carbohydrate,” “Amino-Acids & Derivatives,” and “Nucleoside & Nucleotides” via small organic molecules uptake/utilization/recycling (oligo/monosaccharides, amino-acid degradation, nucleotide conversion, Fig. 2c). Although reduced compared to the control, respiration data showed active utilization of all tested substrates in Cu-exposed plots, including even higher usage of citrate in the hotspot (Fig. 1b, Additional file 1: Table S4). We hypothesized that this may potentially be the consequence of active phage synthesis in Cu-plots, resulting in subsequent release of small molecules from host dead cells that can be readily used by opportunistic heterotrophic generalists. Such phenomenon, called “viral-shunt”, was proposed in marine microbiology/biogeochemistry [56], theorizing that phage-mediated lysis releases organic matter recycled directly by heterotrophs into a “microbial loop” instead of reaching higher trophic levels. Constantly facing the entangled Cu and phage threats, we hypothesized that surviving bacteria may have opted for opportunistic/scavenging strategies to thrive, competing to access easily degradable substrates at the expense of other key functional contributions (e.g., tapping into more complex resources), thus leading to the observed carbon and nitrogen accumulation at the contamination hotspot (Additional file 1: Table S1).

Unfortunately, despite these intriguing observations, testing these hypotheses remains extremely challenging, and our descriptive study design certainly does not allow to investigate such questions in further details. Additional analyses are required to investigate host/phage taxonomy and relationship as well as potential consequences on bacterial adaptation, as transduction mechanisms may become the main driver of bacterial evolution under extreme conditions [66].

### Cu altered the microbial capacity to react to environmental fluctuations

**Fig. 4:**
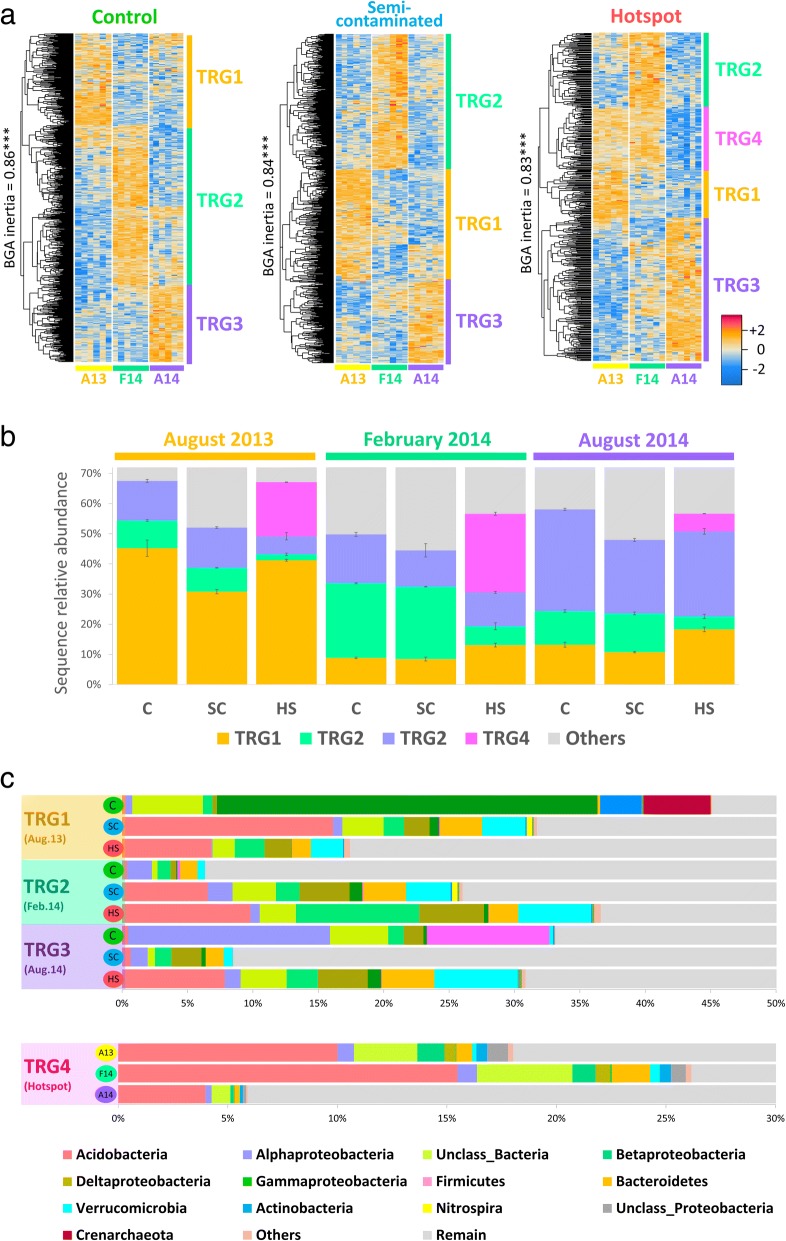
Definition, abundance, and phylogenetic composition of microbial temporal response groups (TRGs). Panel **a** shows the statistical definition and validation of the TGRs in each plot. OTUs with significant temporal response were extracted in each Cu plots (nbGLM, LRT, FDR-corrected *p* < 0.05), and grouped using hierarchical clustering. Validation of TRGs was done with a Between Group Analysis (BGA) and Monte-Carlo simulations with 100,000 group permutations using all OTUs to reinforce randomization power. Panel **b** shows the relative abundance of TRGs in each plot according to sampling time, while panel **c** displays the phylogenetic composition of TRGs

**Fig. 5:**
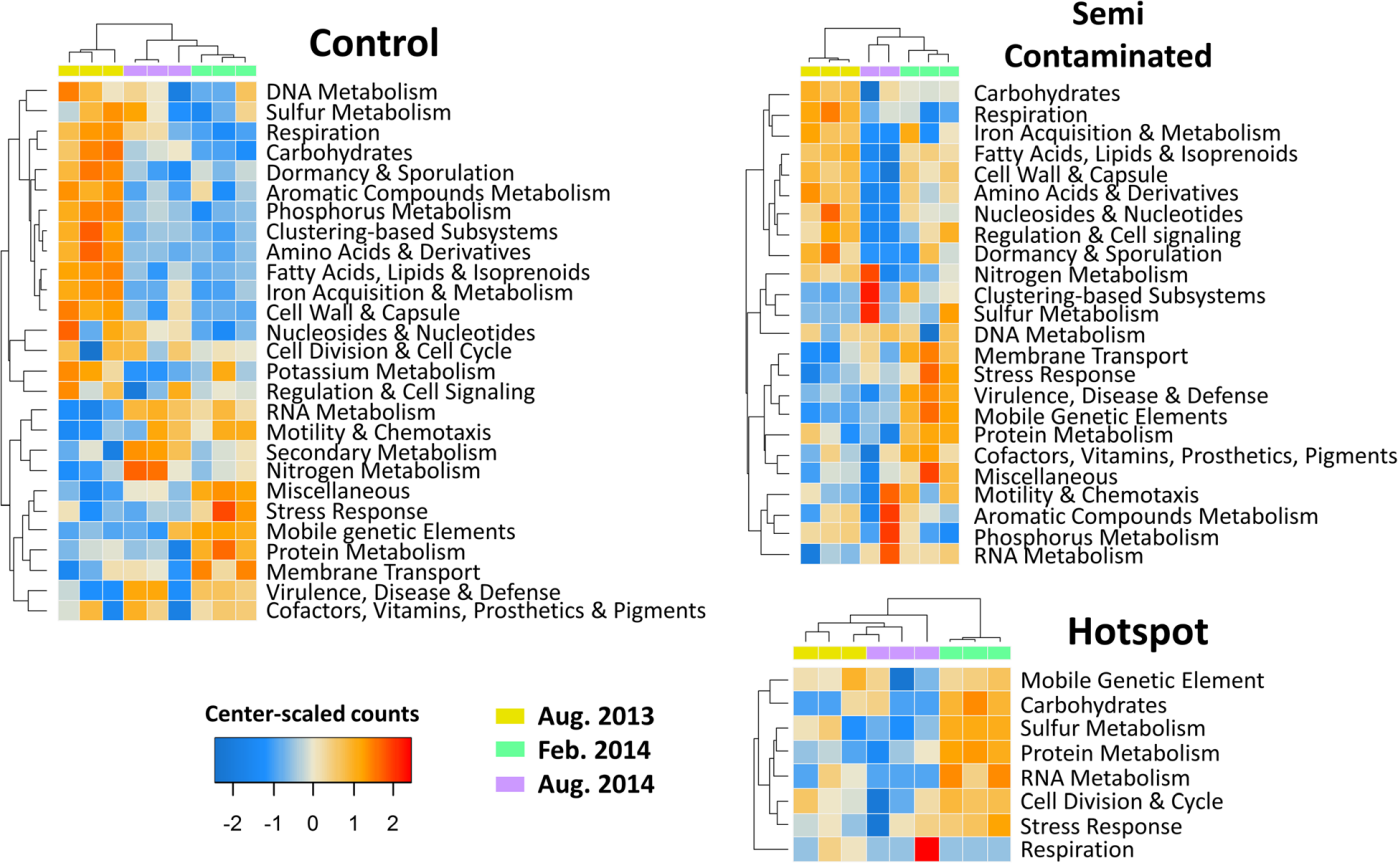
Heatmaps of metatranscriptomic metabolic categories with significant temporal response in each plot. Functions significantly altered were extracted (nbGLM, LRT, FDR correction, *p* < 0.05), summed and plotted in heatmaps as center-scaled mRNA counts values per rows (red nuance = two standard deviations above average, blue nuance = two standard deviations below average)

In this section, we did not intended to conduct a descriptive study of seasonal effects, as this would not be possible with only three time points. Our goal was to look how long-term Cu pollution has affected the capacity of the soil microbial communities to react to environmental fluctuations. Indeed, these environmental fluctuations between the three sampling campaigns were almost as important as Cu in discriminating mRNA profiles, with marked differences between sampling times (Fig. 1b). To evaluate the capacity of the community to react to these environmental fluctuations, we established pairwise comparisons between the three sampling time points (August 2013 vs February 2014; August 2013 vs August 2014; February 2014 vs August 2014) for the 16S rRNA gene transcripts (identification of TRGs; Fig. 4) and metatranscriptomic profiles (Fig. 5) in each plot independently.

**Fig. 6:**
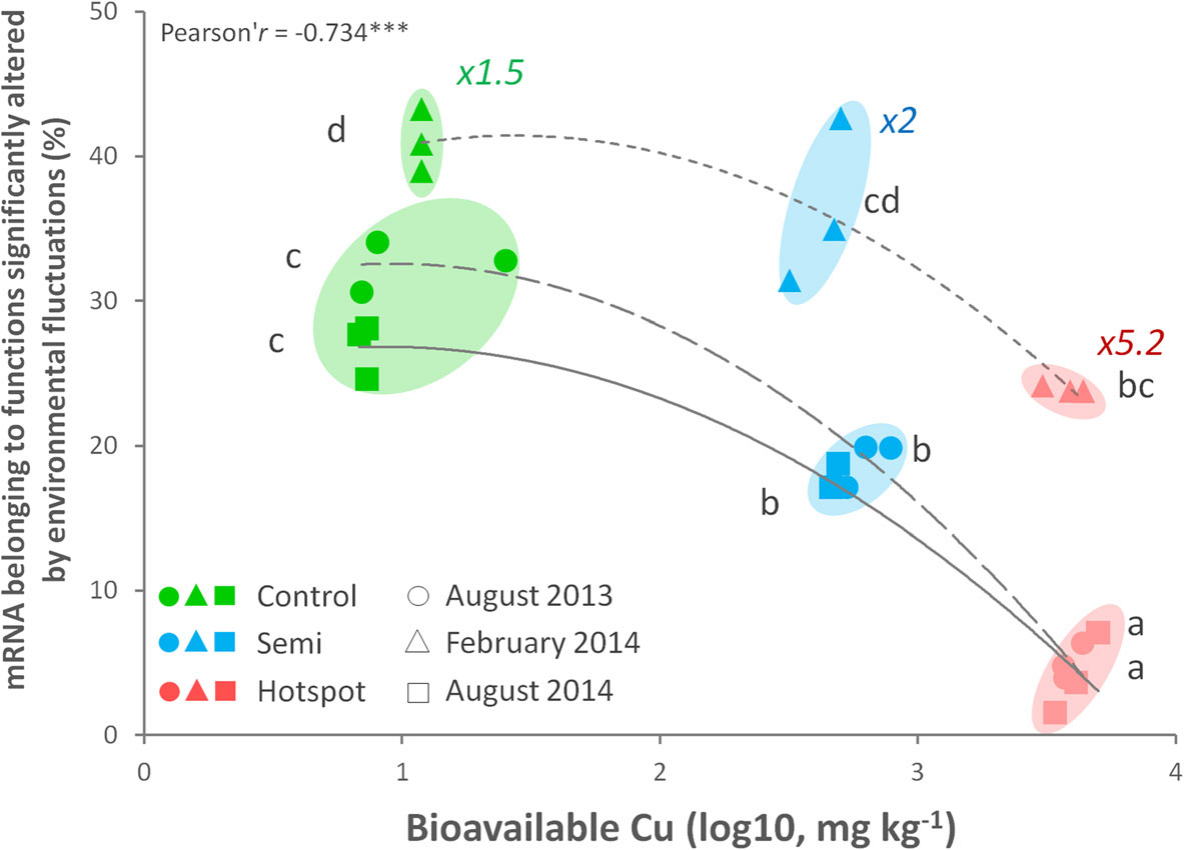
Cu-mediated decoupling of the temporal microbial mRNA response. The *x*-axis gives the bioavailable Cu quantified in each soil samples. The *y*-axis represents the sum of mRNA sequences in each corresponding metatranscriptomic sample belonging to SEED functions that were significantly altered by environmental fluctuation via pairwise comparison between time points (in percentage of total annotated sequences, excluding phages). The decrease of temporally affected mRNA functions along increasing bioavailable Cu (log10, mg kg^−1^) was inferred using the Pearson correlation coefficient (*r* = − 0.73***, *p* = 2.0E−5). Statistical differences between time points in each plot were inferred with ANOVA (Tukey’s HSD post hoc test, *p* < 0.05). Letters are attributed in ascending order, “a” being the smallest average. Different letters indicate statistically significant differences (*p* < 0.05). The colored numbers indicates the observed increase folds in mRNA sequences counts in February 2014 against both August time points

A clear “Cu-decoupling effect” was observed on metatranscriptomes, as temporal response was significantly lower in Cu-plots relative to the control both in terms of abundance of significantly responding functions and their diversity (Fig. 5). At the abundance level, the amount of mRNA sequences belonging to functions significantly altered by sampling time could be displayed in relation to the amount of bioavailable Cu in each samples, yielding a strong negative correlation (Pearson’ *r* = − 0.73, *p* = 2E−5, Fig. 6). This decoupling was also noticed when looking at correlations between key soil parameters, which were progressively lost with elevated Cu concentrations (Additional file 1: Table S5). As expected, the control plot displayed clear temporal patterns with 874 OTUs (58.4 ± 2%) responding into three different TRGs (Fig. 4a), and with 309 functions distributed among 27 metabolic categories being temporally activated (Fig. 5). However, as Cu selected for a less diverse, phylogenetically restrained community, the relationships between microbial activity and soil abiotic factors were lost (Additional file 1: Table S5). This had direct consequences on transcription profiles, which were severely restrained by Cu, especially in the hotspot (Figs. 5 and 6). Concomitantly, the TRG analysis also revealed intense phylogenetic restructuration of temporally responding microbes due to Cu (Fig. 4). Overall, this indicates that Cu-decoupling between time points and microbial activity is concomitantly due to (i) a reduction of the initial genetic/functional diversity pool, not compensated by functional redundancy and long-term adaptation, as well as (ii) a deep phylogenetic reorganization of TRGs.

August time points were expected to have a higher microbial activity, as organic matter decomposition in temperate soils is positively correlated with temperature, regulating catabolic enzymes like glycoside hydrolases [67]. The increase of several key metabolic pathways in the control during August 2013 confirmed it (Fig. 5), correlating with higher respiration levels (Fig. 1b, Additional file 1: Table S4) and phylogenetic/functional diversity observed (Fig. 1b), despite lowered OTU richness/evenness (August 2013, Additional file 1: Figure S1). Indeed, OTUs enhanced in August 2013 were diverse, including Gammaproteobacteria, Crenarchaeota, and Actinobacteria (TRG1, ~ 45%, Fig. 4b, c). This underlines the link between phylogenetic and functional diversity, and their importance over mere OTU richness in maintaining soil functions. Gammaproteobacteria (*Pseudomonas* sp.), Crenarchaeota (Thermoprotei), and Actinobacteria (Actinomycetales) were prevalent in August 2013, being coherent with their ability to cope with drier conditions due to lowered water content [68–70]. Year-to-year analysis revealed similarities between wetter/colder August 2014 and February 2014, correlating with a phylogenetic shift to Alphaproteobacteria (Rhizobiales) and Firmicutes (Bacillales) (TRG3, ~ 35%, Fig. 4b, c). Concomitantly, a drop in “Dormancy/Sporulation” and an upregulation of nitrogen cycling (mostly ammonium assimilation and denitrification), RNA metabolism, motility and secondary metabolism functions between August 2013 and 2014 occurred (Fig. 5). Microbial successions may explain these changes, as Rhizobiales and dormant Bacillales members were presumably reactivated by higher moisture [71, 72].

The response of the semi-contaminated plot in August was intermediate, with significantly less temporally affected mRNA compared to the control (Fig. 6). Still, a preserved functional core including respiration, nitrogen, carbohydrate, and phosphorous metabolism was activated (Fig. 5). Metatranscriptome profiles from August 2014 were highly heterogeneous, including a sample with very low sequence counts and hence not shown (Aug14_SC6, Additional file 1: Table S3). In the hotspot, the observed temporal mRNA response was the smallest (Fig. 6), with no obvious increase in specific functions unlike other plots (Fig. 5). This lack of temporal pattern is mainly due to steadily expressed functions regardless of time, likely being maintained to cope with Cu. The reduced plant cover and root-released photosynthetic substrates may explain this activity disruption [15, 18, 19]. Conversely, functions with highly heterogeneous profiles were observed, like “Respiration” (Fig. 5), highlighting the deleterious effect of Cu on microbial activity and mirroring the heterogeneous distribution of Cu precipitates in this soil (see field pictures, Additional file 1: Figure S2). The phylogenetic composition of August TRGs changed (Fig. 4), as dominating responders were reduced/replaced in Cu-plots. Gammaproteobacteria (Pseudomonadales, TRG1), Crenarchaeota (Thermoprotei, TRG1), Alphaproteobacteria (Rhizobiales, TRG3), and Firmicutes (Bacillales, TRG3) were replaced by Nitrospira (TRG1), Betaproteobacteria (TRG1/3), Alphaproteobacteria (Rhodospiralles, TRG3), Deltaproteobacteria (Myxococcales, TRG3), and Acidobacteria (Gp1/3/6/12/16, TRG3). Some Nitrospira members are known to harbor efficient transporters/efflux systems [73], potentially conferring a selective advantage in Cu-plots during August 2013, filling empty niches abandoned by Cu-sensitive taxa. Myxococcales are also known for their tolerance toward metals [74]. Betaproteobacteria are renowned soil opportunists/copiotrophs [24] active during favorable conditions [75] and suited to conquer empty niches in Cu-plots due to their reported metal-resistance [76]. Finally, an additional strategy appeared in the hotspot: TRG4, being promoted both in August 2013/February 2014 and dominated by Acidobacteria (Fig. 4), reinforcing the idea of a strong Cu-driven disruption and readjustment of the community to environmental fluctuations.

In February, the transcriptional response was the highest in all plots, with respectively 1.5 (control), 2 (semi-contaminated), and 5.2-folds (hotspot) more mRNA sequences significantly enriched compared to August (Fig. 6). This was concomitant to a transient α-diversity increase in richness/evenness (Additional file 1: Figure S1) correlating with water content in all sites (Additional file 1: Table S5). As previously reported, RNA/protein metabolism increased in cold conditions [15], here together with membrane transport, virulence/defense mechanisms, cofactors/vitamins/pigments synthesis, stress response, and MGEs (phages) indicating an activity shift (Fig. 5). This correlated with a well-defined microbial response (TRG2, Fig. 4), featuring cold-associated groups like Bacteroidetes, Verrucomicrobia, and Acidobacteria (Gp6) [77, 78]. Semi-contaminated plot mRNA profiles resemble the control, with enhancement for some functions initially upregulated in the control in August 2013 (e.g., “Cell-wall/Capsule,” “Regulation/Cell-Signaling,” “Amino Acid, Fatty-Acid/Lipids/Isoprenoids,” and “Iron metabolisms,” Fig. 5). This trend was reinforced in the hotspot, where all promoted activities occurred in February. Indeed, while enhanced in other plots in August, cell divisions/cycle and carbohydrate/sulfur metabolisms shifted to February in the hotspot alongside RNA/protein metabolism, phage and stress response (Fig. 5). Overall, these observations suggest a lesser negative effect of Cu during February, allowing some microbial functioning. Since temperature is known to modulate Cu toxicity on this site [79], we hypothesized that cold weather and in situ trapping of bioavailable Cu in ice was responsible for this unexpected activity gain (see field pictures, Additional file 1: Figure S2). This is supported by the hotspot-specific TRG4, only active during February and drier August 2013, but not in the wetter August 2014 (Fig. 4), suggesting the importance of the water status in Cu-plots. While similar in the control and semi-contaminated plots, February-associated TRG2 was significantly reduced in abundance in the hotspot (Fig. 4), and the novel dominant TRG4 strategy appeared, mainly made of Acidobacteria (Gp 3/6/12/16), Unclassified Bacteria, Proteobacteria, and Bacteroidetes, while Verrucomicrobia was reduced (Fig. 4b, c). Like in August, Acidobacteria members also dominated in February in Cu-plots. Acidobacteria are often positively correlated with low pH and nutrient concentration, which was the opposite here, as the hotspot has higher pH, carbon and nitrogen (Additional file 1: Table S1). Although Cu-enriched subdivision Gp6/16 favored high pH, this is not the case for Gp1/3/12 [80], which were abundant in the hotspot (Additional file 1: Table S2). Acidobacteria have reported resistance to metals [81] and close association to edaphic parameters [82], with also unexpected functional versatility still poorly explored [83], especially on elusive subdivisions (e.g., Gp 12/16). Cu-selection of Gp 1/3 is compatible with their reported heterotrophic/generalist lifestyles [83] reinforcing the idea of functionally redundant competing Acidobacteria selected in Cu-plots [25, 84]. Therefore, we hypothesized that the Cu-mediated restructuring of microbial TRGs left vacant niches available to versatile generalist/opportunistic Acidobacteria groups.

Protein metabolism remained one of the few activities maintained despite high Cu concentrations (Fig. 5), correlating with the steady presence of Bacteroidetes in TRG2/4, known for their activity in such conditions [15] and complex molecule degradation capacities (e.g., proteins/polysaccharides) [85, 86]. The higher pH and nitrogen content observed in the hotspot could result from long-term ammonium accumulation from protein degradation and lowered microbial activity in August (Figs. 3 and 5). Concomitant with the higher microbial activity and diversity, we also observed an enrichment of phage mRNA during February in all plots (MGE, Fig. 5), suggesting again that environmental conditions were more favorable during February as opposed to August. In summary, despite lowered microbial and functional response caused by elevated Cu concentration, microbial activity was maintained in February. This functioning upsurge despite the deep phylogenetic restructuring of TRGs has to be discussed in connection with the potential role of phages, which is known to interfere with functional stability despite taxonomic variability [65].

## Conclusion

In this study, besides new knowledge gathered on soil microbial gene expression under long-term Cu pollution, we revealed a marked effect of Cu on the capacity of microbial communities to react to environmental changes relative to the control plot. Metatranscriptomes correlated with both phylogenetic responses through TRGs and recorded soil/microbial properties, being yet still rarely observed [12]. Unlike in August, February had the highest mRNA functional response and OTU diversity in all plots, with specific functions that were maintained regardless of the pollution status. Cu concentration correlated with enhanced phage signatures in mRNA pools, which were even more enriched in February. Cold temperature, soil ice, and phages seemed to play important roles in stabilizing community functioning despite deleterious Cu effects.

## Methods

### Site description and sampling

The experimental field (Hygum, Denmark, 55° 46′ N, 9° 27′ E) is dedicated for research on long-term Cu pollution [87]. Initial pollution originated from wood impregnation activities between 1911 and 1924 [88]. A gridding-mapped gradient was established, where Cu concentrations vary 200~300-folds, with minor differences for other elements [6, 87–89]. Three 16-m^2^ plots corresponding to contrasting contamination levels were sampled within the gradient, a control plot with ambient concentrations (C, ≈ 15 ± 0.5 mg Cu kg^−1^), a semi-contaminated plot (SC, ≈ 450 ± 3.9 mg Cu kg^−1^), and a hotspot (HS, ≈ 4500 ± 292 mg Cu kg^−1^, Additional file 1: Table S1). To assess temporal fluctuations within each plot, we focused on three sampling campaigns showing contrasted environmental conditions: August 2013 (relatively dry/warm), February 2014 (relatively cold/dry), and August 2014 (relatively mild/wet). Weather and soil characteristics are provided in Additional file 1: Table S1. Data gathered for August 2013 (this study) were obtained as described previously [1]. Six representative samples per plot (4–14 cm) were obtained according to standard procedures [90] for each sampling campaign (6 samples × 3 plots × 3 campaigns = 54 samples, Additional file 1: Table S6). Each sample consisted of six subsamples collected at six randomly picked spots within each plot. Samples were manually homogenized, and 2 g was stored in 5 mL of ice-cold LifeGuard Soil Preservation Solution (MOBIO Laboratories, Carlsbad, CA, USA) for RNA extraction.

### 16S rRNA gene transcript amplicon sequencing

To support mRNA findings (this study), 16S rRNA gene transcript profiles from February/August 2014 were integrated [1]. Additionally, year-to-year fluctuations were considered by adding new samples from August 2013 (this study). RNA isolation, cDNA synthesis, cDNA-based 16S rRNA gene amplicon sequencing, quality trimming, OTU definition, and annotation of samples from August 2013 were done as previously described [1]. Sequencing was done following best practices guidelines [91]. The ≈ 460 bp fragment covering the hyper-variable regions V3-V4 of the 16S rRNA gene was amplified with primers 341F (5′GTGCCAGCMGCCGCGGTAA-3′) and 806R (5′GGACTACHVGGGTWTCTAAT-3′) (Sigma-Aldrich, Brøndby, Denmark) from 1:10 cDNA dilutions, tagged and sequenced using 2 × 250 bp paired-end high-throughput Illumina MiSeq Reagent Kits v2 and Illumina® MiSeq® platform (Illumina, San Diego, CA, USA). Sample description is provided in Additional file 1: Table S6.

### 16S rRNA gene transcript amplicon analysis

Based on 16S rRNA-based rarefaction curves (Additional file 1: Figure S3), statistical analysis was done on rarefied contingency tables at *n* = 19,000 counts using the *Rgui* software [92] for generation of α-diversity (Additional file 1: Figure S1) and redundancy analysis (RDA, after log10 transformation). Phylogenetic relatedness between OTUs was assessed with unifrac-based Mean Pairwise Distance index (MPD, Additional file 1: Table S1) using the *picante* package [93]. Major phylogenetic changes at phylum/class levels were assessed by ANOVA (Tukey’s HSD post hoc test, *p* < 0.05, Additional file 1: Table S2). Temporally responding OTUs were extracted with the *edgeR* package [94] using likelihood ratio test after generalized linear modeling using negative binomial distribution (nbGLM LRT, FDR-corrected *p* < 0.05). This method accurately extracts significant OTUs by minimizing false discovery errors [95]. Temporal response groups (TRGs) aggregating OTUs based on their activity patterns were identified using hierarchical clustering and Monte-Carlo simulation as previously described [2, 20]. One thousand five hundred seventy-six OTUs were clustered in four non-randomly defined TRGs, representing ~ 14% of the total OTU richness and 45–70% of the reads. The four TRGs are defined as follows: TRG1 (enhanced in August 2013), TRG2 (enhanced in February 2014), TRG3 (enhanced in August 2014), and TRG4 (unique to the hotspot, enhanced in both August 2013 and February 2014, and lowered in August 2014).

### Metatranscriptome generation

Based on extraction quality, three samples of total RNA extracts from August 2013, 2014 and February 2014 were selected for mRNA sequencing (3 samples × 3 plots × 3 campaigns = 27 samples, Additional file 1: Table S3). rRNA depletion was done after 16S rRNA gene amplicon sequencing was performed, using the Ribo-Zero™ kit with bacterial rRNA removal reagents and the Magnetic Core kit (Epicenter®, Illumina®, WI, USA). Depleted samples were purified using RNeasy® MiniElute® kit (Quiagen®, Copenhagen, Denmark), and ScriptSeq v2 RNA-seq libraries were constructed (Epicenter®, Illumina®, WI, USA). Sequencing was done at the Danish National High-Throughput DNA Sequencing Center using 2 × 150 bp Illumina® HiSeq® Rapid Paired End run. Output information of metatranscriptomic samples in provided in supporting data (Additional file 1: Table S3).

### Metatranscriptome analysis

rRNA reads were filtered using *SortMeRNA* version 2.0 [96]. Filtered mRNA reads were cleaned and assembled using *Biopieces* (http://www.biopieces.org, removal of reads < 50 bp; 20 bp minimum overlap with maximum 40% mismatch). PhiX sequences were trimmed using *Usearch* to ensure correct detection of phage-related sequences. Cleaned mRNA sequences were submitted to MG-RAST for functional annotations [97]. Sequences were not assembled into contigs to avoid the problem of chimera formation arising from highly diverse communities, as previously recommended [98]. Assembled sequences per sample ranged between 2,668,139 and 8,898,760, of which 53–75% obtained predictions among MGRAST databases (Additional file 1: Table S3). After filtering against SEED subsystem hierarchical protein classification using representative hit annotations and improved cut-off parameters (*E*-value< 1.E−10, identity > 70%, alignment > 30 aa), annotated reads were kept for statistical analysis, being coherent with state-of-the-art reported results [15, 26]. While lowering the fraction of workable annotated reads, this conservative method prevents potential misinterpretation arising from unappropriated database matches and wrong assignments [27]. The SEED subsystem hierarchical protein classification was selected for annotations, as it gave the highest annotation results compared to other sources when applying similar stringency cut-off (e.g., KO and COG, see Additional file 1: Table S3). Sample “Aug14_SC6” resulted in very low sequence counts and was not used (Additional file 1: Table S3). Functions responding significantly to time or Cu were extracted (nbGLM LRT, FDR-corrected *p* < 0.05) using the *edgeR* package [94]. Metatranscriptomic profiles were discriminated with a RDA plot, and up and downregulation of genes was assessed with pairwise ratios between plots. Temporal changes in mRNA functions were displayed in individual heatmaps for each plot and as a function of bioavailable Cu.

### MicroResp™ and PLFA analysis

MicroResp™ and phospholipid fatty acid (PLFA) profiles from August 2013 samples (this study) were obtained as previously described [1, 99]. Sieved soil samples (2 mm) were adjusted to 50% of water-holding capacity and pre-incubated according to guidelines (http://www.microresp.com). Seven carbon substrates were used during 6 h of incubation: D(+) Galactose (GAL), L-Malic Acid (MAL), Gamma Amino Butyric Acid (GABI), n-Acetyl Glucosamine (AGL), D(+) Glucose (GLU), Alpha Ketogluterate (AKET), and Citric Acid (CIT). The absorbance of the detection gel in the top micowell plate was recorded (590 nm, Chameleon FP plate reader; Hidex, Turku, Finland) and used to calculate respired CO_2_ (using a standard calibration procedure with known respiration rates) as μg C–CO_2_ g^−1^ dry soil h^−1^. Results are presented in Additional file 1: Table S4 and summarized in Additional file 1: Table S1. For PLFAs, 10 g of dry soil were placed in Teflon centrifuge tubes (Nalge Nunc, Oak Ridge, TN, USA) and extracted in 10 mL of dichloromethane/methanol/citrate buffer (0.15 M; pH 4.0; 1:2:0.8,vol:vol:vol). Supernatants from two repeated extractions were pooled and separated in an organic solvent phase and an aqueous phase by addition of dichloromethane and citrate buffer. Polar lipids in organic solvent phases were purified and derivatized, followed by analysis by gas chromatography and phospholipid-based taxonomic affiliation. Results are given in nmol g^−1^ soil and presented in Additional file 1: Table S7 and summarized in Additional file 1: Table S1. PLFA and MicroResp™ were analyzed using ANOVA to test the effects of copper and sampling time, followed by a Tukey’s HSD post-hoc test (p < 0.05).

## Additional file

Additional file 1: **Figure S1.** Alpha-diversity analysis (richness and Shannon index) of 16S rRNA gene transcript amplicon profiles (cDNA) and metatranscriptomes in the three copper plots at different times (A13: August 2013; F14: February 2014; A14: August 2014). **Figure S2.** Picture of the Hygum site in winter during the February 2014 sampling campaing. **Figure S3.** Rarefaction curves obtained from 16S rRNA gene transcript amplicon profiles (cDNA) and metatranscriptomes in the three copper plots at different time points. **Table S1.** Description of the Hygum plots location, soil characteristics and weather information (average ± SEM, *n* = 18). **Table S2.** RNA-based taxonomic composition of the soil microbiomes depending on copper legacy and sampling time using 16S rRNA gene transcript amplicon sequencing. **Table S3.** Description of the metatranscriptomes generated in this study. **Table S4.** MicroResp™ results summary. **Table S5.** Decoupling of temporal correlations between tested parameters linked to Cu. **Table S6.** Sample description (season and copper doses), nomenclature and total number of 16S rRNA gene transcript sequences assembled. **Table S7.** PLFA results summary. (DOCX 6356 kb)

## Abbreviations

(c)DNA: (Coding) deoxyribonucleic acid
(m/r)RNA: (Messenger/ribosomal) ribonucleic acid
AGL: n-Acetyl glucosamine
AKET: Alpha ketogluterate
CIT: Citric acid
Cu: Copper
GABI: Gamma amino butyric acid
GAL: D(+) Galactose
GLU: D(+) Glucose
MAL: l-Malic acid
MGE(s): Mobile genetic element(s)
MPD: Mean pairwise distance
OTU(s): Operational taxonomic unit(s)
PLFA: Phospholipid and fatty acid
TRG(s): Temporal response group(s)
