# Supplementary material for: Long-term soil metal exposure impaired temporal variation in microbial metatranscriptomes and enriched active phages
Source: Microbiome. 2018 Dec 13;6:223. doi: 10.1186/s40168-018-0606-1 (PMC6292020; doi:10.1186/s40168-018-0606-1)
Supplement: Supplementary file 1 — Figure S1. Alpha-diversity analysis (richness and Shannon index) of 16S rRNA gene transcript amplicon profiles (cDNA) and metatranscriptomes in the three copper plots at different times (A13: August 2013; F14: February 2014; A14: August 2014). Figure S2. Picture of the Hygum site in winter during the February 2014 sampling campaing. Figure S3. Rarefaction curves obtained from 16S rRNA gene transcript amplicon profiles (cDNA) and metatranscriptomes in the three copper plots at different time points. Table S1. Description of the Hygum plots location, soil characteristics and weather information (average ± SEM, n = 18). Table S2. RNA-based taxonomic composition of the soil microbiomes depending on copper legacy and sampling time using 16S rRNA gene transcript amplicon sequencing. Table S3. Description of the metatranscriptomes generated in this study. Table S4. MicroResp™ results summary. Table S5. Decoupling of temporal correlations between tested parameters linked to Cu. Table S6. Sample description (season and copper doses), nomenclature and total number of 16S rRNA gene transcript sequences assembled. Table S7. PLFA results summary. (DOCX 6356 kb) [file 40168_2018_606_MOESM1_ESM.docx]

**Supporting Figures & Tables File**

From Jacquiod *et al.* 2018.

Long-term soil metal exposure impaired temporal variation in microbial metatranscriptomes and enriched active phages. *Microbiome* (under review)

**Figure S1:** Alpha-diversity analysis (richness and Shannon index) of 16S rRNA gene transcript amplicon profiles (cDNA) and metatranscriptomes in the three copper plots at different times (A13: August 2013; F14: February 2014; A14: August 2014). Richness is expressed in OTU numbers for 16S rRNA amplicon, and SEED functions for mRNA profiles, respectively. Statistical differences were inferred from ANOVA (Tukey’s HSD post-hoc test, *p* < 0.05, average ± SEM, n = 6). Letters are attributed in ascending order, “a” being the lowest average. Different letters indicate statistically significant differences.


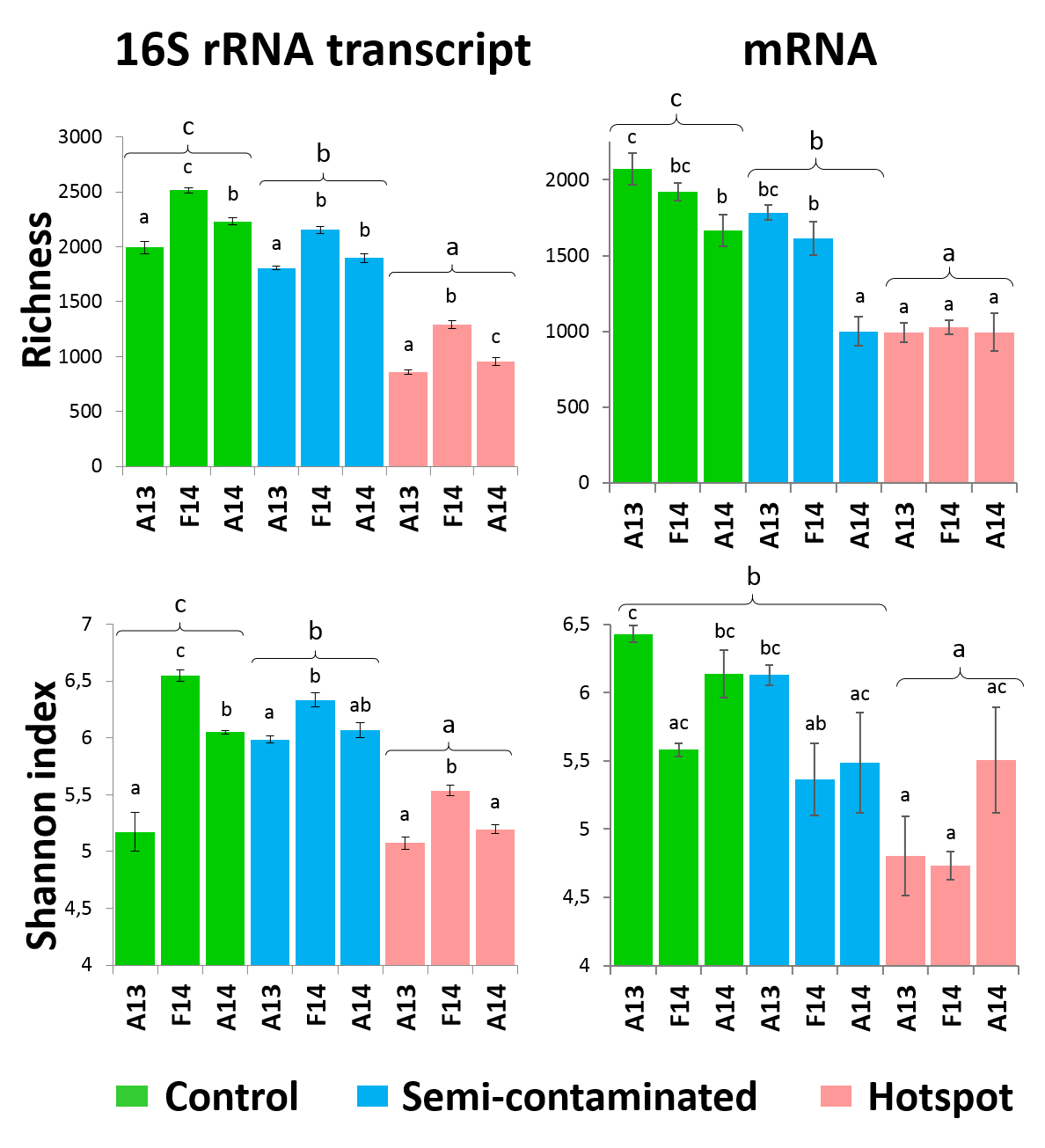


**Figure S2**: Picture of the Hygum site in winter during the February 2014 sampling campaing. Although atmospherical temperature where slightly above 0°C (Tab.S1), the region has been exposed to freezing temperature days before the sampling campaign. Picture a) shows the frozen snow cover of the control plot, picture b shows the frozen soil in the hotspot (down to 20-30cm deep), with close up pictures of frozen copper precipitate/crystals (picture c) and hybernating/frozen ants awaken by the shovel sampling (picture d).


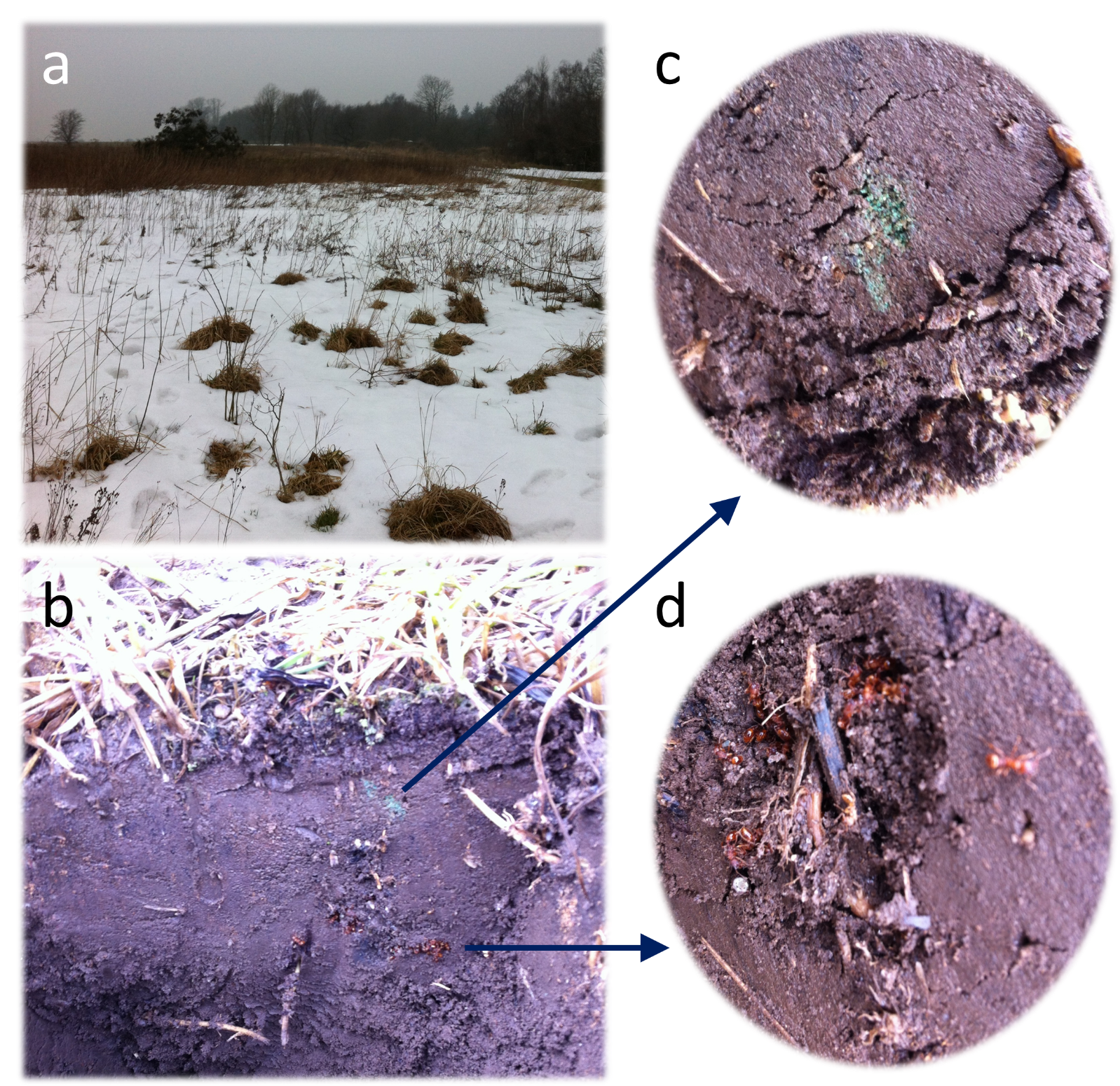


**Figure S3**: Rarefaction curves obtained from 16S rRNA gene transcript amplicon profiles (cDNA) and metatranscriptomes in the three copper plots at different time points.


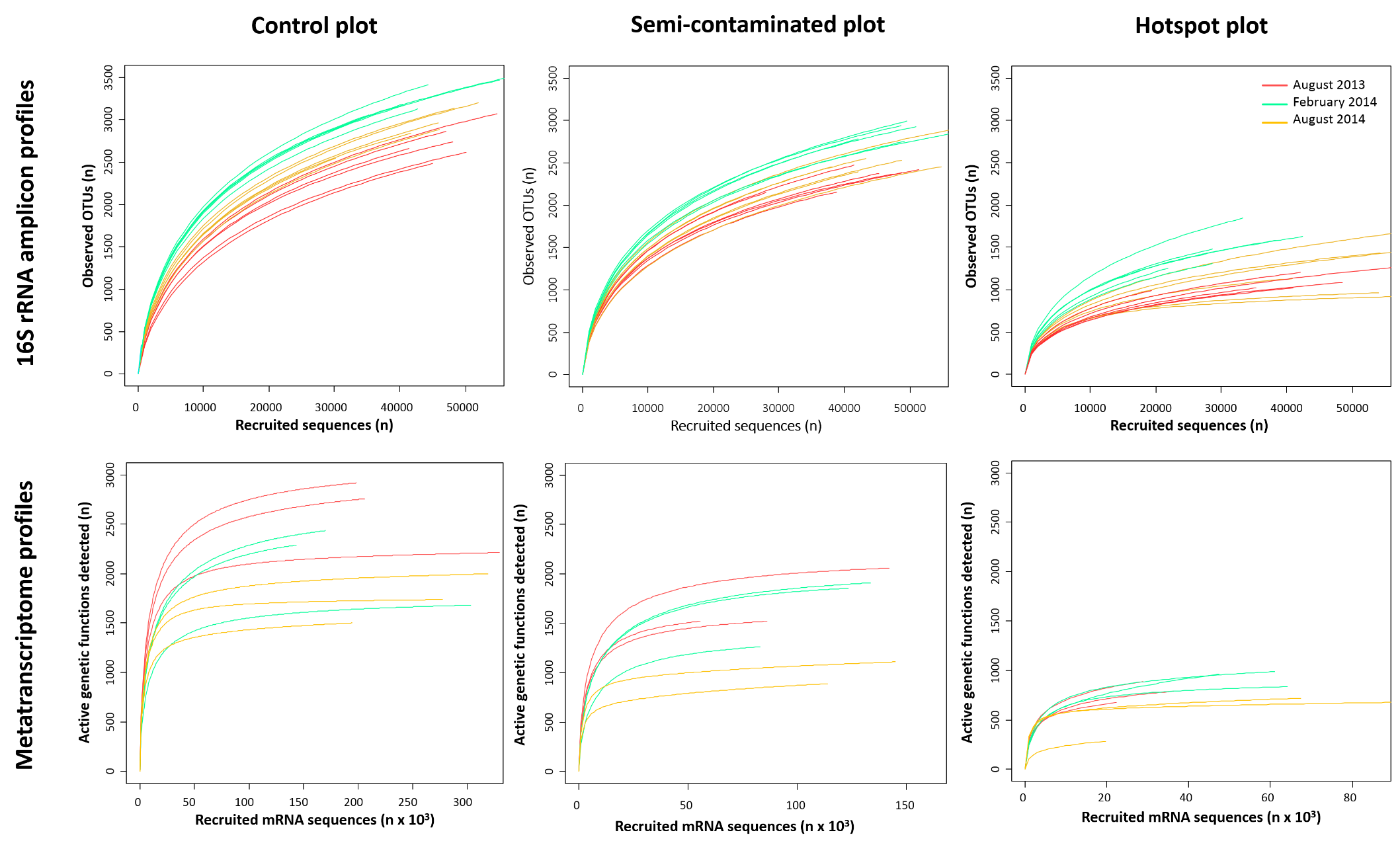


**Table S1**: Description of the Hygum plots location, soil characteristics and weather information (average ± SEM, n = 18). Statistical differences were inferred from ANOVA (Tukey’s HSD post-hoc test, *p* < 0.05). Letters are attributed in ascending order, “a” being the lowest average. Different letters indicate statistically significant differences. The top part provides information when averaging all sampling time points by Cu-plots. Site coordinates (transversal and longitudinal) corresponding to a 4x4 m permanent grid in the Hygum site are given, as well as soil characteristics for each Cu-plot (total Cu, bioavailable Cu, total carbon, total nitrogen, pH, moisture) and microbial properties (total of summed MicroResp^TM^ substrates, fungal and bacterial PLFA fractions and the Z-score of the Mean Pairwise Distance phylogenetic relatedness in the UNIFRAC tree, or MPD). The MPD Z-score inversely varies with the phylogenetic diversity (the higher the diversity, the lower the relatedness). The bottom part of the table provides weather and temporal associated data when averaging all plots by sampling time. Temperature was obtained from the Billund airport meteorological station (ca. 18 km W/SW), while precipitation was obtained from the Jelling meteorological station (ca. 3 km S). All the meteorological data was kindly provided by the Danmarks Meteorologisk Institut (DMI; [www.dmi.dk](http://www.dmi.dk)).

| **The Hygum site: Soil and microbial characteristics in each sampled plot** | | | | |
| --- | --- | --- | --- | --- |
| **Site characteristics** | Plot | Control | Semi-Contaminated | Hotspot |
|  | Plot code | C | SC | HS |
|  | Trans. coordinates (m) | 28-32 | 28-32 | 28-32 |
|  | Long. coordinates (m) | 124-128 | 90-64 | 44-48 |
| **Soil characteristics** | Total Cu (mg kg^-1^) | 15 ±0.49^a^ | 458 ±3.92^b^ | 4526 ±292^c^ |
|  | Bio. Cu (mg kg^-1^) | 0.01 ±1E-3^a^ | 0.5 ±0.02^b^ | 4.42 ±0.16^c^ |
|  | Total C (%) | 2.12 ±0.09^a^ | 2.31 ±0.15^a^ | 3.02 ±0.08^b^ |
|  | Total N (%) | 0.15 ±0.01^a^ | 0.15 ±0.01^a^ | 0.23 ±0.01^b^ |
|  | pH | 6.23 ±0.03^a^ | 6.16 ±0.05^a^ | 6.43 ±0.02^b^ |
|  | Moisture (%) | 20.82 ±0.89^a^ | 22.2 ±0.78^ab^ | 25.1 ±0.65^b^ |
| **Microbial characteristics** | MicroResp (µg CO_2_-C g^-1^ h^-1^) | 12.66 ±0.67^c^ | 9.0 ±0.52^b^ | 6.93 ±0.54^a^ |
|  | PLFA - Fungi (nmol g^-1^) | 1.18 ±0.05^c^ | 0.66 ±0.04^b^ | 0.46 ±0.04^a^ |
|  | PLFA - Bacteria (nmol g^-1^) | 24.85 ±0.95^c^ | 16.98 ±0.88^b^ | 13.88 ±0.83^a^ |
|  | MPD (Z-score) | -3.13 ±0.29^b^ | -3.51 ±0.24^ab^ | -4.08 ±0.11^a^ |
| **The Hygum site: Weather, soil and microbial characteristics for each sampling time** | | | | |
| **Weather characteristics** | Year | 2013 | 2014 | 2014 |
|  | Month | August | February | August |
|  | Sampling time code | A13 | F14 | A14 |
|  | Average temperature (°C) | 18.9 ±1.07^c^ | -0.8 ±1.05^a^ | 17.0 ±0.52^b^ |
|  | Average precipitation (mm) | 0.4 ±0.18^a^ | 0.5 ±0.25^a^ | 5.2 ±2.06^b^ |
| **Soil characteristics** | pH | 6.28 ±0.03^ab^ | 6.17 ±0.04^a^ | 6.46 ±0.05^b^ |
|  | Moisture (%) | 15.78 ±0.81^a^ | 26.0 ±0.52^b^ | 23.27 ±0.69^b^ |
| **Microbial characteristics** | MicroResp (µg CO2-C g^-1^ h^-1^) | 12.92 ±1.04^c^ | 8.25 ±0.57^ab^ | 8.86 ±0.62^ab^ |
|  | PLFA - Fungi (nmol g^-1^) | 0.6 ±0.08^a^ | 0.87 ±0.08^ab^ | 0.96 ±0.12^b^ |
|  | PLFA - Bacteria (nmol g^-1^) | 17.57 ±1.6^a^ | 21.21 ±1.6^a^ | 18.44 ±2^a^ |
|  | MPD (Z-score) | -1.53 ±0.31^b^ | -3.8 ±0.13^a^ | -4.13 ±0.16^a^ |

**Table S2:** RNA-based taxonomic composition of the soil microbiomes depending on copper legacy and sampling time using 16S rRNA gene transcript amplicon sequencing. Taxa are provided at the phylum level, and further detailed at the class level for Proteobacteria and Acidobacteria. Abundances are given in average percentage of reads (± SEM, n = 18). Statistical differences were inferred by ANOVA (by rows, Tukey’s HSD post-hoc test, *p* < 0.05). Letters are attributed in ascending order, “a” being the lowest average. Different letters indicate statistically significant differences.

|  | **Copper legacy effect** | | |  | **Seasonal fluctuations** | | |
| --- | --- | --- | --- | --- | --- | --- | --- |
| **Phylogenetic group** | **Control** | **Semi** | **Hotspot** |  | **August 2013** | **February 2014** | **August 2014** |
| Proteobacteria | 53.56 ±1.43^c^ | 42.96 ±1.62^b^ | 38.46 ±1.65^a^ |  | 42.95 ±2.81^a^ | 40.93 ±1.48^a^ | 51.09 ±0.88^b^ |
| - Alphaproteobacteria | 16.65 ±1.15^ab^ | 18.61 ±1.21^b^ | 15.83 ±1.05^a^ |  | 12.78 ±0.45^a^ | 15.41 ±0.45^b^ | 22.9 ±0.8^c^ |
| - Betaproteobacteria | 8.98 ±0.43^a^ | 9.74 ±0.38^a^ | 11.06 ±0.85^b^ |  | 9.99 ±0.78^ab^ | 9.05 ±0.49^a^ | 10.75 ±0.5^b^ |
| - Gammaproteobacteria | 15.63 ±2.69^c^ | 4.3 ±0.58^b^ | 1.79 ±0.35^a^ |  | 11.47 ±3.34^b^ | 4.43 ±0.67^a^ | 5.83 ±0.75^a^ |
| - Deltaproteobacteria | 10.68 ±0.64^b^ | 9.38 ±0.53^a^ | 8.7 ±0.44^a^ |  | 7.71 ±0.31^a^ | 10.66 ±0.55^b^ | 10.39 ±0.55^b^ |
| Acidobacteria | 10.95 ±0.73^a^ | 20.72 ±0.58^b^ | 30.45 ±1.07^c^ |  | 17.74 ±1.8^a^ | 24.19 ±2.04^b^ | 20.19 ±1.99^ab^ |
| - Gp3 | 3.38 ±0.17^a^ | 10.71 ±0.5^b^ | 15.89 ±0.72^c^ |  | 8.89 ±1.15^a^ | 9.33 ±1.14^ab^ | 11.76 ±1.52^b^ |
| - Gp6 | 2.88 ±0.33^a^ | 2.86 ±0.28^a^ | 2.86 ±0.38^a^ |  | 2.32 ±0.1^b^ | 4.65 ±0.18^c^ | 1.64 ±0.1^a^ |
| - Gp16 | 0.9 ±0.09^a^ | 1.09 ±0.11^a^ | 5.94 ±0.55^b^ |  | 2.7 ±0.57^b^ | 3.72 ±0.82^b^ | 1.51 ±0.33^a^ |
| - Gp1 | 0.82 ±0.05^a^ | 2.5 ±0.14^b^ | 2.97 ±0.22^b^ |  | 1.62 ±0.19^a^ | 2.1 ±0.25^ab^ | 2.57 ±0.3^b^ |
| - Gp4 | 1.12 ±0.17^c^ | 0.77 ±0.13^b^ | 0.09 ±0.02^a^ |  | 0.5 ±0.08^a^ | 1.22 ±0.2^b^ | 0.27 ±0.05^a^ |
| - Gp2 | 0.3 ±0.03^a^ | 1.2 ±0.09^b^ | 0.3 ±0.07^a^ |  | 0.41 ±0.12^a^ | 0.61 ±0.13^ab^ | 0.78 ±0.11^b^ |
| - Gp12 | 0.05 ±0.01^a^ | 0.13 ±0.01^b^ | 0.27 ±0.03^c^ |  | 0.1 ±0.02^a^ | 0.14 ±0.01^a^ | 0.22 ±0.04^b^ |
| Firmicutes | 5.54 ±1.05^a^ | 5.13 ±0.64^a^ | 5.32 ±0.93^a^ |  | 4.72 ±0.73^a^ | 3.97 ±0.53^a^ | 7.3 ±1.1^b^ |
| Bacteroidetes | 5.18 ±0.42^a^ | 4.15 ±0.7^a^ | 4.47 ±0.54^a^ |  | 3.1 ±0.22^a^ | 7.31 ±0.52^b^ | 3.39 ±0.26^a^ |
| Verrucomicrobia | 5.68 ±0.67^c^ | 4.24 ±0.49^b^ | 1.88 ±0.2^a^ |  | 2.17 ±0.23^a^ | 6.26 ±0.71^c^ | 3.36 ±0.3^b^ |
| Actinobacteria | 3.11 ±0.35^b^ | 1.56 ±0.2^a^ | 1.75 ±0.24^a^ |  | 3.3 ±0.36^b^ | 1.66 ±0.14^a^ | 1.46 ±0.21^a^ |
| Nitrospira | 0.21 ±0.02^a^ | 0.95 ±0.13^b^ | 2.75 ±0.58^c^ |  | 2.56 ±0.62^b^ | 0.74 ±0.15^a^ | 0.6 ±0.08^a^ |
| Crenarchaeota | 1.85 ±0.58^c^ | 0.46 ±0.15^b^ | <0.01 ± <0.01^a^ |  | 2.15 ±0.55^b^ | 0.07 ±0.02^a^ | 0.09 ±0.03^a^ |
| Chloroflexi | 0.12 ±0.01^c^ | 0.08 ±0.01^b^ | 0.01 ±0.002^a^ |  | 0.05 ±0.01^a^ | 0.07 ±0.01^a^ | 0.08 ±0.02^a^ |
| Planctomycetes | 0.07 ±0.01^c^ | 0.04 ±0.01^b^ | 0.02 ±0.003^a^ |  | 0.05 ±0.01^b^ | 0.06 ±0.01^b^ | 0.01 ±0.003^a^ |
| Unclassified | 12.83 ±0.32^a^ | 19.17 ±1.42^b^ | 14.54 ±1.39^a^ |  | 20.54 ±1.54^b^ | 13.96 ±0.45^a^ | 12.05 ±0.69^a^ |

**Table S3**: Description of the metatranscriptomes generated in this study. The table includes the sampling time (Aug.13: August 2013, Aug.14: August 2014; Feb.14: February 2014), copper (C: Control, SC: Semi-contaminated, HS: Hotspot), total assembled sequences, fractions with predicted hits in MGRAST resources (Pred.), failed quality control (Fail.), rRNA sequences, predicted hits in SEED, COG and KO databases. Metatranscriptomes are publically available at the MG-RAST platform (http://metagenomics.anl.gov/).

| **MGRAST name** | **Accession** | **Sample** | **Season** | **Copper** | **Total** | **Pred. (%)** | **Fail. (%)** | **rRNA (%)** | **SEED (%)** | **COG %** | **KO %** |
| --- | --- | --- | --- | --- | --- | --- | --- | --- | --- | --- | --- |
| Aug13_C4_assemble_pairs_all.fna | 4669638.3 | Aug13_C4 | Aug.13 | C | 2668139 | 86.79 | 9.90 | 11.47 | 8,75 | 6.5 | 6.15 |
| Aug13_C5_assemble_pairs_all.fna | 4669634.3 | Aug13_C5 | Aug.13 | C | 2794041 | 87.07 | 10.44 | 10.41 | 8,48 | 6.37 | 6 |
| Aug13_C6_assemble_pairs_all.fna | 4669633.3 | Aug13_C6 | Aug.13 | C | 8377868 | 86.20 | 10.44 | 12.28 | 8,1 | 6.1 | 6.04 |
| Aug13_HS1_assemble_pairs_all.fna | 4669623.3 | Aug13_HS1 | Aug.13 | HS | 2956198 | 86.37 | 8.33 | 16.76 | 1,01 | 0.49 | 0.55 |
| Aug13_HS3_assemble_pairs_all.fna | 4669615.3 | Aug13_HS3 | Aug.13 | HS | 3924373 | 83.78 | 8.28 | 16.49 | 1 | 0.53 | 0.58 |
| Aug13_HS4_assemble_pairs_all.fna | 4669621.3 | Aug13_HS4 | Aug.13 | HS | 4274174 | 83.82 | 9.71 | 16.29 | 1,15 | 0.43 | 0.49 |
| Aug13_SC1_assemble_pairs_all.fna | 4669631.3 | Aug13_SC1 | Aug.13 | SC | 4992640 | 82.13 | 8.73 | 11.71 | 3,6 | 2.69 | 3.15 |
| Aug13_SC3_assemble_pairs_all.fna | 4669613.3 | Aug13_SC3 | Aug.13 | SC | 4656590 | 82.33 | 8.68 | 11.50 | 2,48 | 1.87 | 2.28 |
| Aug13_SC6_assemble_pairs_all.fna | 4669628.3 | Aug13_SC6 | Aug.13 | SC | 3023340 | 82.51 | 8.71 | 10.45 | 2,51 | 1.67 | 1.95 |
| Aug14_C2_assemble_pairs_all.fna | 4669620.3 | Aug14_C2 | Aug.14 | C | 7804301 | 85.31 | 8.60 | 10.87 | 5 | 3.85 | 4.32 |
| Aug14_C4_assemble_pairs_all.fna | 4669639.3 | Aug14_C4 | Aug.14 | C | 8479966 | 85.32 | 8.87 | 11.19 | 4,12 | 3.24 | 3.67 |
| Aug14_C6_assemble_pairs_all.fna | 4669632.3 | Aug14_C6 | Aug.14 | C | 5091474 | 84.93 | 9.82 | 10.81 | 4,85 | 3.04 | 3.38 |
| Aug14_HS1_assemble_pairs_all.fna | 4669629.3 | Aug14_HS1 | Aug.14 | HS | 8747745 | 94.03 | 5.97 | 8.65 | 1,21 | 0.24 | 0.29 |
| Aug14_HS2_assemble_pairs_all.fna | 4669619.3 | Aug14_HS2 | Aug.14 | HS | 8898006 | 87.04 | 7.63 | 11.92 | 2,12 | 1.58 | 1.68 |
| Aug14_HS4_assemble_pairs_all.fna | 4669618.3 | Aug14_HS4 | Aug.14 | HS | 7803449 | 78.51 | 8.78 | 14.18 | 0,45 | 0.7 | 0.69 |
| Aug14_SC1_assemble_pairs_all.fna | 4669637.3 | Aug14_SC1 | Aug.14 | SC | 8734814 | 79.21 | 8.89 | 10.71 | 2,33 | 1.45 | 1.61 |
| Aug14_SC4_assemble_pairs_all.fna | 4669614.3 | Aug14_SC4 | Aug.14 | SC | 6554468 | 75.11 | 9.50 | 10.64 | 2,6 | 1.29 | 1.39 |
| Aug14_SC6_assemble_pairs_all.fna | 4669640.3 | Aug14_SC6 | Aug.14 | SC | 64913 | 79.84 | 2.16 | 7.19 | 10,32 | 0.38 | 0.41 |
| Feb14_C4_assemble_pairs_all.fna | 4669635.3 | Feb14_C4 | Feb.14 | C | 5180833 | 85.85 | 8.56 | 10.35 | 3,87 | 2.69 | 2.88 |
| Feb14_C5_assemble_pairs_all.fna | 4669626.3 | Feb14_C5 | Feb.14 | C | 4384229 | 85.31 | 8.30 | 10.30 | 3,81 | 2.51 | 2.79 |
| Feb14_C6_assemble_pairs_all.fna | 4669630.3 | Feb14_C6 | Feb.14 | C | 4347253 | 86.05 | 8.15 | 10.61 | 3,87 | 2.7 | 3.11 |
| Feb14_HS1_assemble_pairs_all.fna | 4669622.3 | Feb14_HS1 | Feb.14 | HS | 5191829 | 85.17 | 8.43 | 16.01 | 1,51 | 0.92 | 0.97 |
| Feb14_HS2_assemble_pairs_all.fna | 4669617.3 | Feb14_HS2 | Feb.14 | HS | 4222184 | 85.17 | 9.65 | 15.74 | 1,45 | 0.85 | 0.96 |
| Feb14_HS4_assemble_pairs_all.fna | 4669627.3 | Feb14_HS4 | Feb.14 | HS | 4080946 | 83.72 | 11.02 | 15.16 | 2,03 | 1.08 | 1.13 |
| Feb14_SC1_assemble_pairs_all.fna | 4669616.3 | Feb14_SC1 | Feb.14 | SC | 4687840 | 85.04 | 8.12 | 10.45 | 3,2 | 2.16 | 2.38 |
| Feb14_SC2_assemble_pairs_all.fna | 4669636.3 | Feb14_SC2 | Feb.14 | SC | 5090520 | 81.74 | 7.97 | 10.60 | 2,11 | 1.29 | 1.4 |
| Feb14_SC4_assemble_pairs_all.fna | 4669625.3 | Feb14_SC4 | Feb.14 | SC | 4268373 | 82.95 | 8.46 | 10.06 | 3,88 | 2.45 | 2.77 |

**Table S4**: MicroResp^TM^ results summary. Respiration results are given in μg C-CO2 g^−1^ dry soil h^−1^ for the following substrates: D(+) Galactose (GAL), L-Malic Acid (MAL), Gamma Amino Butyric Acid (GABI), n-Acetyl Glucosamine (AGL), D(+) Glucose (GLU), Alpha Ketogluterate (AKET) and Citric Acid (CIT). Statistical differences within each substrate were inferred from ANOVA (by columns, Tukey’s HSD post-hoc test, *p* < 0.05). Letters are attributed in ascending order, “a” being the lowest average. Different letters indicate statistically significant differences. Results were either averaged by plots and sampling time, and nested sampling times within each plots.

| **Tested conditions** | | **GAL** | **MAL** | **GABI** | **AGL** | **GLU** | **AKET** | **CIT** |
| --- | --- | --- | --- | --- | --- | --- | --- | --- |
| **Averaged**  **by plot (n = 18)** | **Control** | 1.14 ±0.09^c^ | 1.7 ±0.12^b^ | 0.63 ±0.06^c^ | 0.99 ±0.08^c^ | 1.78 ±0.13^c^ | 3.83 ±0.28^b^ | 1.99 ±0.16^a^ |
|  | **Semi-Contaminated** | 0.55 ±0.05^b^ | 1 ±0.08^a^ | 0.32 ±0.03^b^ | 0.69 ±0.06^b^ | 1.07 ±0.09^b^ | 2.65 ±0.16^a^ | 2.01 ±0.15^a^ |
|  | **Hotspot** | 0.23 ±0.03^a^ | 1.13 ±0.1^a^ | 0.14 ±0.02^a^ | 0.25 ±0.03^a^ | 0.52 ±0.05^a^ | 2.26 ±0.15^a^ | 2.33 ±0.15^a^ |
| **Averaged by**  **sampling time (n = 18)** | **August 2013** | 0.84 ±0.14^b^ | 1.63 ±0.15^b^ | 0.47 ±0.07^b^ | 0.78 ±0.12^a^ | 1.43 ±0.19^b^ | 3.75 ±0.28^b^ | 2.68 ±0.14^b^ |
|  | **February 2014** | 0.54 ±0.09^a^ | 1.04 ±0.08^a^ | 0.34 ±0.05^ab^ | 0.55 ±0.08^a^ | 0.93 ±0.11^a^ | 2.31 ±0.17^a^ | 1.7 ±0.1^a^ |
|  | **August 2014** | 0.54 ±0.08^a^ | 1.15 ±0.09^a^ | 0.29 ±0.05^a^ | 0.6 ±0.08^a^ | 1 ±0.13^ab^ | 2.68 ±0.19^a^ | 1.94 ±0.11^a^ |
| **August 2013**  **(n = 6)** | **Control** | 1.55 ±0.32^d^ | 2.3 ±0.29^c^ | 0.84 ±0.18^e^ | 1.31 ±0.33^d^ | 2.45 ±0.26^e^ | 5.06 ±0.53^d^ | 2.64 ±0.51^bc^ |
|  | **Semi-Contaminated** | 0.69 ±0.18^bc^ | 1.21 ±0.2^ab^ | 0.37 ±0.04^bd^ | 0.76 ±0.22^c^ | 1.26 ±0.22^cd^ | 3.42 ±0.48^bc^ | 2.63 ±0.35^bc^ |
|  | **Hotspot** | 0.29 ±0.2^a^ | 1.38 ±0.65^b^ | 0.18 ±0.16^ab^ | 0.26 ±0.22^a^ | 0.59 ±0.28^ab^ | 2.77 ±0.9^ac^ | 2.77 ±0.87^c^ |
| **February 2014**  **(n = 6)** | **Control** | 0.96 ±0.31^c^ | 1.3 ±0.35^ab^ | 0.54 ±0.23^d^ | 0.78 ±0.31^c^ | 1.38 ±0.35^cd^ | 2.81 ±1.13^ac^ | 1.51 ±0.58^a^ |
|  | **Semi-Contaminated** | 0.45 ±0.21^ab^ | 0.78 ±0.27^a^ | 0.33 ±0.13^ad^ | 0.59 ±0.33^ac^ | 0.88 ±0.36^ac^ | 2.18 ±0.29^a^ | 1.6 ±0.4^a^ |
|  | **Hotspot** | 0.22 ±0.08^a^ | 1.06 ±0.11^ab^ | 0.15 ±0.04^ab^ | 0.29 ±0.1^ab^ | 0.54 ±0.14^a^ | 1.96 ±0.11^a^ | 1.99 ±0.15^ac^ |
| **August 2014**  **(n = 6)** | **Control** | 0.92 ±0.14^c^ | 1.49 ±0.2^b^ | 0.51 ±0.14^cd^ | 0.88 ±0.15^c^ | 1.52 ±0.14^d^ | 3.61 ±0.5^c^ | 1.82 ±0.27^ab^ |
|  | **Semi-Contaminated** | 0.51 ±0.25^ab^ | 1.02 ±0.4^ab^ | 0.26 ±0.17^abc^ | 0.71 ±0.26^bc^ | 1.07 ±0.44^bcd^ | 2.36 ±0.5^ab^ | 1.79 ±0.53^ab^ |
|  | **Hotspot** | 0.18 ±0.1^a^ | 0.95 ±0.17^ab^ | 0.09 ±0.05^a^ | 0.21 ±0.11^a^ | 0.42 ±0.16^a^ | 2.07 ±0.26^a^ | 2.23 ±0.52^ac^ |

**Table S5**: Decoupling of temporal correlations between tested parameters linked to Cu. Table shows the evolution of Pearson correlation coefficient (r) and associated p-value (p) in the different plots along the Cu concentration gradient. The temporal information from the three sampled time points were used to generate each correlation. MPD: mean pairwise distance. *, **, and *** indicate *p* < 0.05, *p* < 0.01 and *p* < 0.001, respectively.

| **Copper plot** | **Control** | | **Semi-contaminated** | | **Hotspot** | |
| --- | --- | --- | --- | --- | --- | --- |
| **Pearson's correlation** | ***r*** | ***p*** | ***r*** | ***p*** | ***r*** | ***p*** |
| Respiration vs MPD | 0.75*** | 4.9E-6 | 0.51** | 3.7E-3 | 0.35 | 0.08 |
| Respiration vs pH | 0.36* | 0.05 | 0.14 | 0.46 | -0.11 | 0.55 |
| Respiration vs OTU Richness | -0.79*** | 5.1E-7 | -0.26 | 0.17 | -0.32 | 0.11 |
| Respiration vs Moisture | -0.87*** | 2.4E-6 | -0.61** | 7.5E-3 | -0.47* | 0.05 |
| Moisture vs MPD | -0.89*** | 2.2E-10 | -0.69*** | 2.1E-5 | -0.25 | 0.22 |
| Moisture vs pH | -0.49** | 6.2E-3 | 0.10 | 0.60 | -0.11 | 0.57 |
| Moisture vs OTU Richness | 0.83*** | 4.5E-8 | 0.51** | 4.4E-3 | 0.70*** | 4.1E-5 |

**Table S6**: Sample description (season and copper doses), nomenclature and total number of 16S rRNA gene transcript sequences assembled. Amplicon sequencing was deposited at the Sequence Read Archive database (SRA) under accession PRJNA414414 (https://www.ncbi.nlm.nih.gov/bioproject/PRJNA414414/).

| **Replicates** | **Year** | **Month** | **Copper plot** | **Code** | **Counts** |
| --- | --- | --- | --- | --- | --- |
| Aug13.C1 | 2013 | August | Control | A13C | 44966 |
| Aug13.C2 | 2013 | August | Control | A13C | 50106 |
| Aug13.C3 | 2013 | August | Control | A13C | 48044 |
| Aug13.C4 | 2013 | August | Control | A13C | 41374 |
| Aug13.C5 | 2013 | August | Control | A13C | 54770 |
| Aug13.C6 | 2013 | August | Control | A13C | 47046 |
| Aug13.SC1 | 2013 | August | Semi-Contaminated | A13SC | 51430 |
| Aug13.SC2 | 2013 | August | Semi-Contaminated | A13SC | 48756 |
| Aug13.SC3 | 2013 | August | Semi-Contaminated | A13SC | 45253 |
| Aug13.SC4 | 2013 | August | Semi-Contaminated | A13SC | 41471 |
| Aug13.SC5 | 2013 | August | Semi-Contaminated | A13SC | 38821 |
| Aug13.SC6 | 2013 | August | Semi-Contaminated | A13SC | 27961 |
| Aug13.HS1 | 2013 | August | Hotspot | A13HS | 48480 |
| Aug13.HS2 | 2013 | August | Hotspot | A13HS | 19325 |
| Aug13.HS3 | 2013 | August | Hotspot | A13HS | 35343 |
| Aug13.HS4 | 2013 | August | Hotspot | A13HS | 40999 |
| Aug13.HS5 | 2013 | August | Hotspot | A13HS | 62121 |
| Aug13.HS6 | 2013 | August | Hotspot | A13HS | 42086 |
| Feb14.C1 | 2014 | February | Control | F14C | 42753 |
| Feb14.C2 | 2014 | February | Control | F14C | 35209 |
| Feb14.C3 | 2014 | February | Control | F14C | 55214 |
| Feb14.C4 | 2014 | February | Control | F14C | 58432 |
| Feb14.C5 | 2014 | February | Control | F14C | 40403 |
| Feb14.C6 | 2014 | February | Control | F14C | 44286 |
| Feb14.SC1 | 2014 | February | Semi-Contaminated | F14SC | 49557 |
| Feb14.SC2 | 2014 | February | Semi-Contaminated | F14SC | 49133 |
| Feb14.SC3 | 2014 | February | Semi-Contaminated | F14SC | 50932 |
| Feb14.SC4 | 2014 | February | Semi-Contaminated | F14SC | 42103 |
| Feb14.SC5 | 2014 | February | Semi-Contaminated | F14SC | 59422 |
| Feb14.SC6 | 2014 | February | Semi-Contaminated | F14SC | 48632 |
| Feb14.HS1 | 2014 | February | Hotspot | F14HS | 33238 |
| Feb14.HS2 | 2014 | February | Hotspot | F14HS | 28718 |
| Feb14.HS3 | 2014 | February | Hotspot | F14HS | 42475 |
| Feb14.HS4 | 2014 | February | Hotspot | F14HS | 38442 |
| Feb14.HS5 | 2014 | February | Hotspot | F14HS | 28628 |
| Feb14.HS6 | 2014 | February | Hotspot | F14HS | 21831 |
| Aug14.C1 | 2014 | August | Control | A14C | 30148 |
| Aug14.C2 | 2014 | August | Control | A14C | 45860 |
| Aug14.C3 | 2014 | August | Control | A14C | 41605 |
| Aug14.C4 | 2014 | August | Control | A14C | 45997 |
| Aug14.C5 | 2014 | August | Control | A14C | 48232 |
| Aug14.C6 | 2014 | August | Control | A14C | 52015 |
| Aug14.SC1 | 2014 | August | Semi-Contaminated | A14SC | 70107 |
| Aug14.SC2 | 2014 | August | Semi-Contaminated | A14SC | 38263 |
| Aug14.SC3 | 2014 | August | Semi-Contaminated | A14SC | 42085 |
| Aug14.SC4 | 2014 | August | Semi-Contaminated | A14SC | 54858 |
| Aug14.SC5 | 2014 | August | Semi-Contaminated | A14SC | 48780 |
| Aug14.SC6 | 2014 | August | Semi-Contaminated | A14SC | 43304 |
| Aug14.HS1 | 2014 | August | Hotspot | A14HS | 66140 |
| Aug14.HS2 | 2014 | August | Hotspot | A14HS | 54257 |
| Aug14.HS3 | 2014 | August | Hotspot | A14HS | 57808 |
| Aug14.HS4 | 2014 | August | Hotspot | A14HS | 40865 |
| Aug14.HS5 | 2014 | August | Hotspot | A14HS | 53986 |
| Aug14.HS6 | 2014 | August | Hotspot | A14HS | 57090 |

**Table S7**: PLFA results summary. Results are given in nmol g^-1^ and for the four different fraction independently (Fungi, Actinobacteria, Gram positive bacteria and Gram negative bacteria). Statistical differences within each substrate were inferred from ANOVA (by columns, Tukey’s HSD post-hoc test, *p* < 0.05). Letters are attributed in ascending order, “a” being the lowest average. Different letters indicate statistically significant differences. Results were either averaged by plots and sampling time, and nested sampling times within each plots.

| **Tested conditions** | | **Fungi** | **Actinobacteria** | **Gram positive** | **Gram negative** |
| --- | --- | --- | --- | --- | --- |
| **Averaged by**  **plot (n = 18)** | **Control** | 1.25 ±0.07^b^ | 2.09 ±0.1^b^ | 11.5 ±0.57^b^ | 12.73 ±0.61^b^ |
|  | **Semi-Contaminated** | 0.68 ±0.06^a^ | 0.92 ±0.06^a^ | 8.97 ±0.65^a^ | 7.01 ±0.54^a^ |
|  | **Hotspot** | 0.51 ±0.06^a^ | 0.71 ±0.05^a^ | 7.64 ±0.65^a^ | 5.64 ±0.47^a^ |
| **Averaged by**  **sampling time (n = 18)** | **August 2013** | 0.6 ±0.08^a^ | 1.24 ±0.16^a^ | 8.43 ±0.62^a^ | 7.9 ±0.86^a^ |
|  | **February 2014** | 0.87 ±0.08^ab^ | 1.38 ±0.17^a^ | 10.18 ±0.61^a^ | 9.65 ±0.83^a^ |
|  | **August 2014** | 0.96 ±0.12^b^ | 1.11 ±0.16^a^ | 9.5 ±0.88^a^ | 7.83 ±0.99^a^ |
| **August 2013**  **(n = 6)** | **Control** | 1.04 ±0.05^cd^ | 2.14 ±0.1^b^ | 10.9 ±0.58^ab^ | 12.55 ±0.51^bc^ |
|  | **Semi-Contaminated** | 0.48 ±0.05^ab^ | 0.89 ±0.09^a^ | 8.1 ±0.95^ab^ | 6.1 ±0.64^a^ |
|  | **Hotspot** | 0.29 ±0.03^a^ | 0.69 ±0.07^a^ | 6.28 ±0.67^a^ | 5.05 ±0.53^a^ |
| **February 2014**  **(n = 6)** | **Control** | 1.22 ±0.06^de^ | 2.29 ±0.14^b^ | 12.06 ±0.77^b^ | 13.58 ±0.92^c^ |
|  | **Semi-Contaminated** | 0.8 ±0.06^bc^ | 1.06 ±0.05^a^ | 10.42 ±0.3^ab^ | 8.6 ±0.51^ab^ |
|  | **Hotspot** | 0.59 ±0.1^ab^ | 0.77 ±0.1^a^ | 8.07 ±1.22^ab^ | 6.76 ±0.99^a^ |
| **August 2014**  **(n = 6)** | **Control** | 1.49 ±0.16^e^ | 1.84 ±0.23^b^ | 11.54 ±1.48^b^ | 12.05 ±1.56^bc^ |
|  | **Semi-Contaminated** | 0.76 ±0.13^bc^ | 0.82 ±0.15^a^ | 8.4 ±1.64^ab^ | 6.32 ±1.23^a^ |
|  | **Hotspot** | 0.64 ±0.12^ac^ | 0.67 ±0.09^a^ | 8.57 ±1.33^ab^ | 5.11 ±0.77^a^ |
